# An adipocyte-endothelial framework linking adipose tissue inflammation to cardiometabolic vulnerability in obesity

**DOI:** 10.21203/rs.3.rs-10603589/v1

**Published:** 2026-08-12

**Authors:** Vaishali Chaurasiya, Luyang Li, Aina Lluch, Ana Fernández-Sánchez, Jukka Harju, Dan Duc Pham, Sanni Perttunen, Salla Keskitalo, Markku Varjosalo, Kirsi H. Pietiläinen, P.A. Nidhina Haridas, José M. Moreno-Navarrete, José I. Rodríguez-Hermosa, Encarna Piedrafita-Serra, Asha Kar, Päivi Pajukanta, Markku Laakso, Ville Männistö, Jussi Pihlajamäki, Minna U. Kaikkonen, Gema Frühbeck, Oriol A. Rangel-Zúñiga, Francisco J. Tinahones, Carlos Diéguez, Ralph Burkhardt, Marcus Höring, Gerhard Liebisch, Johanna Pörschke, María Gómez-Serrano, Johannes Graumann, Witold Szymanski, José M. Fernández-Real, You Zhou, Vesa M. Olkkonen, Francisco J. Ortega

**Affiliations:** 1 Minerva Foundation Institute for Medical Research, Biomedicum 2U – Helsinki, Finland; 2 Doctoral Programme in Clinical Research, University of Helsinki – Helsinki, Finland; 3 Division of Infection and Immunity, School of Medicine, Cardiff University – Cardiff, United Kingdom; 4 Systems Immunity Research Institute, Cardiff University – Cardiff, United Kingdom; 5 Girona Biomedical Research Institute – Girona, Spain; 6 CIBER de la Fisiología de la Obesidad y la Nutrición – Madrid, Spain; 7 Department of Gastrointestinal Surgery, Abdominal Center, University of Helsinki and Helsinki University Hospital – Helsinki, Finland; 8 Molecular Systems Biology Research Group & Proteomics Unit, HiLIFE Helsinki Institute of Life Science, Institute of Biotechnology, University of Helsinki – Helsinki, Finland; 9 Obesity Research Unit, Research Program for Clinical and Molecular Metabolism, Faculty of Medicine, Helsinki University Hospital, University of Helsinki – Helsinki, Finland; 10 HealthyWeightHub, Endocrinology, Abdominal Center, Helsinki University Hospital – Helsinki, Finland; 11 School of Medicine, University of Girona – Girona, Spain; 12 Bioinformatics Interdepartmental Program, UCLA – Los Angeles, USA; 13 Institute for Precision Health, David Geffen School of Medicine at UCLA – Los Angeles, USA; 14 Institute of Clinical Medicine, University of Eastern Finland and Kuopio University Hospital – Kuopio, Finland; 15 Institute of Public Health and Clinical Nutrition, University of Eastern Finland and Kuopio University Hospital – Kuopio, Finland; 16 A.I. Virtanen Institute for Molecular Sciences, University of Eastern Finland – Kuopio, Finland; 17 Instituto de Investigación Sanitaria de Navarra (IdISNA), Clínica Universidad de Navarra – Pamplona, Spain; 18 Maimónides Biomedical Research Institute of Córdoba (IMIBIC), Reina Sofia University Hospital, University of Córdoba – Córdoba, Spain; 19 Instituto de Investigación Biomédica de Málaga (IBIMA), Virgen de la Victoria Hospital, University of Málaga – Málaga, Spain; 20 Center for Research in Molecular Medicine and Chronic Diseases (CiMUS, University of Santiago de Compostela, Instituto de Investigación Sanitaria – Santiago de Compostela, Spain; 21 Institute of Clinical Chemistry and Laboratory Medicine, University Hospital Regensburg – Regensburg, Germany; 22 Institute for Tumor Immunology, Center for Tumor Biology and Immunology, Marburg University – Marburg, Germany; 23 Institute of Translational Proteomics & Core Facility, Marburg University – Marburg, Germany; 24 Department of Anatomy, Faculty of Medicine, University of Helsinki – Helsinki, Finland

**Keywords:** Adipose tissue, adipocyte, endothelial cell, proteomics, lipidomics, meta-inflammation, obesity, weight loss

## Abstract

**Background:**

Obesity-related cardiometabolic disease is linked to impaired adipose tissue function, but the underlying molecular programs are difficult to assign to specific adipose-resident cell types, to mechanistically connect to the activation state of macrophages, and to distinguish from alterations that may normalize with weight loss.

**Methods:**

We integrated a layered design combining untargeted proteomics and lipidomics to define obesity-associated, cell-type-resolved molecular phenotypes across adipocytes and adipose microvascular endothelial cells, explore whether an inflammatory milieu reproduces adipose-resident cell dysfunction, and identify features that show evidence of recovery after weight loss. Next, we validated in adipose tissue transcriptomes the elements that exhibit the strongest association with dyslipidaemia, hypertriglyceridemia, and/or hyperglycaemia to identify gene signatures of cardiometabolic relevance.

**Results:**

Adipocytes from subjects with obesity show suppression of mitochondrial energy metabolism together with impaired lipid plasticity, as reflected by triglyceride remodelling. By mimicking an inflammatory milieu with macrophage-conditioned media, we reproduced many of these changes in adipocyte cultures. Endothelial cells exhibited yet another, opposite trajectory in obesity, with reduced cell-cycle signalling and increased mitochondrial activation, which were recapitulated in vitro when these cells were exposed, respectively, to the secretions of inflamed macrophages and adipocytes. Bulk adipose tissue proteomes and lipidomes showed evidence of metabolic improvement after weight loss, including restoration of mitochondrial and substrate-handling pathways alongside reciprocal triglyceride remodelling. Together with inflammation-responsive adipocyte mitochondrial and lipid-handling dysfunction, our cell-type-informed framework probes macrophage and adipocyte-to-endothelial activation in obesity, and delineates cross-context cellular programs associated with weight loss. Notably, when integrated with transcriptomic resources, these layers of information prioritized determinants linked to impaired metabolism, and were used to generate models that can assess cardiometabolic vulnerability in subjects with obesity (AUROC values between 0.88 and 0.99).

**Conclusions:**

Our study reveals adipocyte and endothelial cell-specific elements acting as molecular signatures of adipose tissue inflammation with cardiometabolic implications.

## INTRODUCTION

Obesity is increasingly common worldwide [1] and strikingly associated with an elevated risk of metabolic and cardiovascular disease [2]. A central link between excessive adiposity and cardiometabolic complications is impaired adipose tissue function, mainly characterized by chronic low-grade inflammation interfering with mitochondrial metabolism, lipid storage, endocrine signalling, and adipose tissue homeostasis [3–5]. Importantly, many pathological consequences attributed to obesity are thought to reflect not adipose mass per se, but altered cellular programs within fat depots, particularly in adipocytes and the stromal vascular niche, that can impair adipose synthetic, oxidative, and secretory functions [6]. Indeed, adipose tissue is an essential, complex, and highly dynamic metabolic organ whose capacity to expand, contract, and regulate systemic energy balance is fundamental in both health and disease [7]. Even in the context of the debated concept of “metabolically healthy” obesity [8,9], adipocyte metabolism and endocrine activity can become disrupted, paralleling changes in adipose-resident macrophage activation [10], progenitor cell commitment [11], and endothelial cell (EC) biology [12].

Adipose tissue is highly vascularized. Beyond oxygenation and nutrient/waste transport, blood vessels shape exposure to circulating hormones and growth factors, enable trafficking of immune and stromal cells, and influence fatty acid mobilization, processes integral to adipose metabolic and endocrine functions [13]. Adipose-resident EC themselves participate in lipid handling, energy homeostasis, and metabolic health [14]. Over the last decade, impaired vascular density and EC dysfunction have emerged as hallmarks of excessive adiposity [15]. Vascular adaptation is also tightly coupled to adipose remodelling: adipose expansion depends on angiogenesis [16], while the adipose microenvironment (e.g., hypoxia, inflammatory cues, and paracrine signals from adipose cells) can either promote or blunt EC responses [17,18]. Because the mural cell compartment of adipose vasculature contains progenitor cells that can differentiate into adipocytes [19], the immune-vascular-adipocyte axis is positioned to influence both depot remodelling and cardiometabolic risk.

Despite already available extensive transcriptomic profiling of adipose tissue in obesity, two shortcomings limit physiological interpretation. First, bulk tissue signatures conflate changes in cell-intrinsic programs with shifts in cellular composition, making it difficult to assign key pathways to specific adipose cell types. Second, it remains unclear which obesity-associated molecular alterations represent reversible dysfunction (e.g., programs that recover with weight loss), as opposed to context-specific correlation. Addressing these gaps requires integration across cell types, perturbation models that mimic the inflammatory adipose milieu, and within-person evidence of recovery following clinically meaningful weight loss. Here, we combined flow cytometry-based cell isolation with untargeted, omics-scale proteomic and lipidomic profiling to resolve adipocyte and EC-specific molecular phenotypes across complementary contexts relevant to the pathophysiology of obesity. First, we profiled ex vivo isolated adipocytes and adipose-derived EC from individuals in the lean body weigh range and individuals living with obesity. Then, we modelled obesity-like inflammatory stress in vitro using macrophage-conditioned media (MCM) and adipocyte-conditioned media (ACM) to address adipocyte-to-EC signalling. Next, we assessed recovery-associated remodelling in paired bulk adipose tissue before and after bariatric surgery-induced weight loss. Finally, we integrated these layers of information with several independent transcriptomic resources and cohort data to prioritize robust adipocyte and EC determinants linked to impaired metabolism. Together, our framework identifies non-immune adipose cell-type-informed molecular determinants altered in obesity, reveals which are inflammation-responsive, and delineates adipocyte and EC-linked programs that show evidence of recovery with weight loss, providing molecular signatures of metabolic vulnerability in people with obesity.

## METHODS

### Cohorts.

For the study of *ex vivo* isolated adipocytes and adipose-derived EC (cohort #1), ~5 g of omental adipose tissue were retrieved from 17 patients (2 men and 15 women) undergoing surgical cholecystectomy for gallstone disease at the Department of Gastrointestinal Surgery, Abdominal Center, HUS Jorvi hospital of Helsinki, in Finland (**Table S1**). Upon extraction, adipose samples were transferred to the laboratory and processed under sterile conditions within 24 hours, as explained in reference [20]. In the longitudinal study (cohort #2), abdominal subcutaneous adipose tissue was collected in eighteen (n=18) surgical patients (2 men and 16 women) with severe obesity during and after enduring laparoscopic Roux-en-Y gastric bypass and subsequent weight loss (**Table S2**). Macrobiopsies of adipose tissue were minced in pieces of ~150 mg immediately after extraction, then introduced in sterile containers, snap frozen in liquid nitrogen, and stored at −80°C until further processing. Human samples were handled following standard operating procedures, and information from subjects included in this study was provided by the FATBANK platform, promoted by the CIBEROBN and coordinated by the Biomedical Research Institute of Girona (IDIBGI) Biobank (Biobank IDIBGI), integrated in the Spanish National Biobanks Network (ISCIII, Madrid). All subjects provided written informed consent before entering the study, which was approved by the Ethics Committees for Clinical Investigation (CEIC) of the Biobanc IDIBGI (ethics approval study number B.0000872) and the Helsinki and Uusimaa Hospital District (ethics approval study number HUS/23/2022), respectively. For validation purposes, we reanalysed bulk adipose RNA-seq data available in multiple independent study cohorts. The METabolic Syndrome In Men (METSIM; ethics approval study number 171/2004) sample (cohort #3) consists of 10,197 Finnish males with ages between 45 and 73, recruited in the University of Eastern Finland and Kuopio University Hospital, Kuopio, Finland, as described in reference [21]. The study design was approved by the Ethics Committee of the Northern Savo Hospital District, and all participants gave written informed consent. All research conformed to the principles of the Helsinki Declaration, and bulk RNA-seq data was obtained in the subcutaneous adipose tissue of 335 unrelated participants [22] (**Table S3**). The cohort #4 (Finnish Twin Cohort) consisted of 49 twin pairs discordant for clinical characteristics of obesity (ethics approval study number 270/13/03/01/2008) [23]. The cohort #5 involved 451 obese adults (30.6% men) from the ADIPOMIT cohort [24] (**Table S4**), including patients who had undergone surgical intervention between 2009 and 2020, and allowed the removal of adipose tissue for biomedical research, given written informed consent. The replication analysis in cohort #6 was selected for confirmatory purposes in the bulk RNA-seq of 241 obese adults (30.7% men) from the Kuopio OBesity Surgery (KOBS) cohort [4] (**Table S5**). The ADIPOMIT study (ethics approval study number 2019.062) was approved by the Ethics Committee of the Hospital Dr Josep Trueta of Girona (Spain). The KOBS workflow (ethics approval study numbers 54/2005, 104/2008, and 27/2010) was approved by the Ethics Committee of the Northern Savo Hospital District (Finland).

### Clinical measures.

Weight and height were measured after a 12-hours overnight fast in light clothing. BMI was calculated as weight (in kilograms) divided by height (in meters) squared. Obesity was defined when BMI≥30 kg/m^2^. Blood pressure was measured in the supine position on the right arm after a 10 min rest. A standard sphygmomanometer of appropriate cuff size was used, and the first and fifth phases were recorded. Blood samples were collected following an overnight fast. Samples were analysed at the corresponding core facilities of participating hospitals in Girona and Helsinki, using standardized methods. Concentrations of plasma glucose were measured using the spectrophotometric hexokinase and glucose-6-phosphate dehydrogenase assay in a Beckman Glucose Analyzer 2 (Beckman Coulter). Measures of total cholesterol were obtained by enzymatic methods on a BM/Hitachi 747 analysis system (Roche), and high (HDL) and low (LDL) density lipoprotein cholesterol were quantified after precipitation with polyethylene glycol 6000 at a final concentration of 100 g/l. Blood haemoglobin (HbA1c) was measured during routine laboratory tests, and blood triglycerides were quantified by the reaction of glycerol-phosphate-oxidase and peroxidase on a Hitachi 917 Rack Chemistry Analyzer (Roche). All participants were requested to withhold alcohol and caffeine prior to the analyses.

### Adipose-resident cell isolation.

Adipose tissue was obtained from the intra-abdominal area of gallstone patients undergoing surgical cholecystectomy. The biopsies were immersed in cold phosphate-buffered saline (PBS) containing 2% penicillin/streptomycin and promptly transported to the laboratory on ice. Collected tissue was immediately processed for isolation of adipocytes and adipose-derived microvascular EC. Adipocytes and EC were segregated according to our standardized protocol, as previously described in reference [20]. Briefly, the extracellular matrix was digested in 90 rpm shaking incubator with collagenase II buffer (Gibco, Paisley, UK) for 20–25 min at 37°C. Digested fat solution was gently poured through a sterile 250 μm mesh filter (Sintab, 6111–025043), gently squeezed and further rinsed twice with PBS. The cell suspension was then transferred to a sterile separation funnel and allowed to stand for 3 min. Adipocytes were then collected and incubated for 5 min with 5 ml of 1xRBC lysis buffer (eBioscience, 00–4300-54) to remove red blood cells, further washed twice with PBS, collected to 50 ml falcon tubes from the separation funnel, and centrifuged at 50×g for 3 min. Then, three aliquots of adipocytes (150–300 μl) were snap frozen in liquid nitrogen for multi-omic study preparation. The stromal vascular cells (SVC) were centrifuged at 1,000 rpm for 5 minutes at 4°C, after which the pellet was incubated with 1× RBC lysis buffer and subsequently washed with PBS. The resulting SVC pellet was resuspended in culture medium and transferred to a T-75 culture flask. SVC were cultured and maintained in a 5% CO_2_ incubator at 37°C in a humidified atmosphere, with endothelial cell growth media (PromoCell, C-22011). EC were segregated using fluorescence-activated cell sorting (FACS) with antibodies against positive CD31 and CD144 in CD45 negative SVC, further cultured in ECGM media, and passaged twice before preparing proteomics samples. EC purity and marker expression of cell cultures prepared by using this protocol was optimised and validated in our previous publication [20].

### Cell cultures.

Commercially available cryopreserved preadipocytes obtained in one Caucasian women aged 30–50 years and BMI of 25–30 kg/m^2^ (SP-F-2) were purchased (Zen-Bio, Inc.). To induce the adipogenic conversion, adipocyte progenitors were led to grow in preadipocyte medium (PM-1), then incubated with adipocyte differentiation medium (DM-2) for 7 days. This media is composed of DMEM/Ham’s F-12 (1:1), HEPES, FBS, biotin, pantothenate, insulin, dexamethasone, IBMX, PPARγ agonist, penicillin, streptomycin and amphotericin B. Thereafter, differentiating adipocytes were maintained in adipocyte maintenance (AM-1) media (i.e., DM-2 without IBMX and PPARγ agonists) for 7 days. Preadipocytes were maintained in PM-1 media during the whole process and used as reference control for the process of differentiation. The human monocyte cell line THP-1 (ATCC, TIB-202) was cultured in RPMI 1640 medium (Gibco, 21875–034) containing 10% FBS, 5 mM glucose (Sigma, G8644), 2 mM L-glutamine, 50 mg/ml Gentamicin (Gibco, 15710–064), and 20 mM HEPES. Floating monocytes were then incubated for 24 h with 0.162 mM phorbol 12-myristate 13-acetate (Merck, P1585) to induce the adherent pro-inflammatory type 1 macrophage-like state (M1). M1 macrophages were washed with PBS and incubated 24 h with fresh medium containing 10 ng/ml LPS (Sigma, L4516). The resulting macrophage LPS-conditioned medium (MCM) was collected and centrifuged at 400*g* for 5 min, filtered, diluted in adipocyte media, and applied to adipocyte cultures for 72 hours. Both preadipocytes, differentiated control adipocytes, and adipocytes treated with macrophage media were collected and used for non-targeted proteomics and lipidomics. After 72 hours from the last medium change, the supernatants were collected, centrifuged for 10 min at 1500 rpm, filtered to eliminate any debris, and indicated as adipocyte MCM-conditioned medium (ACM) or control. ACM was stored at −80°C for further experiments. Simpson-Golabi-Behmel syndrome (SGBS) preadipocytes were cultured and differentiated for 14 days using established protocols [25], generating human adipocytes with characteristic morphology and substantial lipid accumulation. SGBS adipocytes responding to 1% MCM were used for mitochondrial stress flux analysis and oxygen consumption in an Agilent Seahorse XF Pro Analyzer (see below). Human adipose microvascular endothelial cells (HAMEC) were purchased (PeloBiotech, Planegg/Martinsried, Germany) and cultured upon arrival in ECGM media with 1x supplement (PromoCell, C-22011). At passage 4, they were incubated for 72 hours with either 1% control media (RPMI with 1% PS and 10% FBS) or 1% MCM, and with 10% ACM or control media. Post-treatment, EC were washed twice with PBS before being lysed by means of freshly prepared RIPA buffer with 1% SDS (MCM-HAMEC), or 8M urea in 50 mM ammonium bicarbonate (ACM-HAMEC).

### Proteomics.

Isolated adipocytes and adipose-resident EC (study #1), and ACM-HAMEC cultures (study #2) were dispatched to the Proteomics Unit of the Institute of Biotechnology at the University of Helsinki for proteomic assessment, using liquid chromatography tandem mass spectrometry (LC-MS/MS) on a Bruker timsTOF Pro. Adipocyte, progenitor cell, and MCM-HAMEC cultures (study #2), as well as bulk adipose tissue samples (study #3) were transferred to the Philipps-Universtität, in Marburg (Germany). Protein amounts were assessed using a Bruker timsTOF Ultra by the Core Facility Translational Proteomics. The protocol for proteomic sample preparation in isolated adipocytes, which are characterized by extreme lipid content, was adapted from the literature [26]. To begin with, 500 μl of proteomic lysis buffer [1% (wt/vol) sodium deoxycholate in 100 mM triethylammonium] was added to flash-frozen adipocytes, which were then lysed using a tissue homogenizer. The lysates were centrifuged at 6,000*g* for 15 min at 4°C. Cleared lysate was carefully removed by a 1 ml syringe and further sonicated and heat incubated at 95°C for 5 minutes. To extract proteins from the lysate, methanol and chloroform extraction was carried out as described [20]. Specifically, protein extraction was done using methanol and chloroform in a 4:1 ratio. 800 μl of methanol was added to 200 μl of protein lysate and vortexed thoroughly, followed by the addition of 200 μl of chloroform. Subsequently, 600 μl of H_2_O was added and the sample was centrifuged for 1 minute at 14,000*g*. After centrifugation, the samples were phase separated into three layers and the top aqueous layer was discarded without disturbing the interphase protein precipitate. Following this, 800 μl of methanol was added to the remaining layers, vortexed, and centrifuged for 5 minutes at 20,000*g*. The supernatant was then discarded, and the protein pellet resuspended into 200 μl of the above protein lysis buffer. In parallel, adipose-derived EC were rinsed with PBS twice, and 140 μl of 8 M urea in 50 mM ammonium bicarbonate buffer were used for lysis. The protein concentration of adipocytes and EC samples was measured using the Pierce^™^ BCA Protein Assay Kit (Thermo Scientific^™^, 23225), according to the manufacturer’s protocol. The proteomic analysis was conducted using 10 μg of adipocyte-derived proteins and 20 μg of sample from EC. Adipocyte samples were brought to 200 μl with 50 mM NH_4_HCO_3_ and EC samples to 150 μl. Next, 5 mM TCEP [Tris(2-carboxyethol) phosphine hydrochloride salt, #C4706 Sigma-Aldrich, USA] was incubated at 37 °C for 30 min to reduce the samples. For alkylation, 10 mM iodoacetamide (Acros Organics, 122271000) was used for 30 minutes at room temperature, and samples were trypsin digested using 1:20 enzyme to protein ratio Sequencing Grade Modified Trypsin (Promega, V5113) for 16 h at 37°C. Further, 10% trifluoroacetic acid (TFA, VWR, 85049.051) was used to acidify the samples. The AC samples were then centrifuged 16,000 xg, 10 min at room temperature to precipitate the sodium deoxycholate in the buffer, and desalting was done for both sample types using BioPureSPN PROTO 300 C18 Mini columns (HUM S18V, Nest Group) according to the manufacturer’s protocol. Thereafter, a centrifuge concentrator (Concentrator Plus, Eppendorf) was used to dry the peptide samples, which were reconstituted in 30 μl buffer A [0.1% TFA, 1% acetonitrile (VWR) in HPLC grade water (Fisher)]. The samples were diluted 1:20 in 0.1 % formic acid in HPLC water and loaded into Evotip Pure (Evosep, EV2011) according to the manufacturer’s protocol. An Evosep One (Evosep) Chromatography system coupled to a hybrid trapped ion mobility quadrupole TOF mass spectrometer with a CaptiveSpray nano-electrospray ion source (Bruker Daltonics) was used to analyze the samples. Mobile phase A (0.1 % formic acid in water) and mobile phase B (0.1 % formic acid in acetonitrile) were used for phase separation in conjunction to an 8 cm × 150 μm column [1.5 μm C18 beads (Evosep, EV1109)]. MS analysis was done using positive-ion mode and MSFragger pipeline (FragPipe analysis platform (version 20.0) with MSFragger (version 3.8)[27], Philosopher (version 5.0.0), and IonQuant (version 1.9.8) for peptide identification using raw (.d) files as input). Uniprot reference human proteome UP000005640 was used as protein sequence database (see in supplemental **Table S6**). For studies #2 and #3, protein lysates were obtained as previously described by Gómez-Serrano *et al*. [28]. Briefly, 80–100 mg of adipose tissue were homogenized in 150 μl of RIPA buffer (Thermo, 89901) supplemented with 1:100 Halt Protease and Phosphatase Inhibitor Cocktail (Thermo, 78442) by using the Sample Grinding Kit (Cytiva, 80–6483-37). Manufacturer’s instructions were slightly adapted to the lipid nature of human fat, including two grinding-centrifugation steps using 2/3 and 1/3 of the lysis volume, respectively. All steps were done at 4°C, including a final additional clean-up centrifugation step of collected protein supernatants. Protein concentration was estimated by Pierce^™^ BCA Protein Assay Kit (Thermo, 23225). Next, protein extracts were prepared for MS-based proteomics using a modified single-pot, solid-phase-enhanced sample preparation (SP3) method [29]. This protocol integrates protein extraction and detergent removal with C18 solid phase extraction (SPE) for peptide desalting, according to an in-house protocol (http://doi.org/10.5281/zenodo.17570946), with minor modifications. 50 μg of protein per sample was used for preparation. The SP3 procedure was performed in 500 μL Eppendorf Tubes. Chromabond C18WP spin columns were utilized for the C18 SPE desalting step. The final peptide concentration was equalized to 25 ng/μL for injection onto the analytical column. Peptide injection, separation and measurement on Bruker timsTOF Ultra as well as subsequent spectrum matching with Dia-NN was performed as described in http://doi.org/10.5281/zenodo.17475274. For study #3 (bulk adipose tissue), sample loading was performed in “oneCol” mode, loading directly onto the analytical column and omitting the trap-column. MS acquisition was operating in “sensitive” mode. For study #3 (adipocytes), sample loading was equally performed in “oneCol” mode, and the gradient extended to 90 min. For HAMEC, the gradient length was shortened to 45 min. Spectrum matching was performed against the Human Uniprot.org database. All databases used are uploaded along with the description to the repository (see below the Data availability section). Downstream data processing and statistical analysis used the Autonomics package developed in-house (Bioconductor: 10.18129/B9.bioc.autonomics, version: 1.17.16, 1.1.7.14, 1.15.145, 1.15.235, or 1.15.345). Proteomic data were filtered to retain proteins with summed MaxLFQ intensity greater than zero across replicates in both the study and control groups. MaxLFQ intensities were subsequently log2 transformed. Raw log2 intensities (studies #1 and #3) or MaxLFQ intensities (Study #2) were used for quantitation. Measurements from only two precursors (Np) per sample were converted to NA (Not Applicable) for that particular sample. Proteins with a q-value of <0.01 were included for further analysis. Differential abundance of protein groups was evaluated using the Limma package (version 3.54.2) [30]. The R version 4.2.3 (https://www.r-project.org/) code used for processing of spectrum matching (FragPipe and Dia-NN) output data and statistical analysis has been uploaded along with the mass spectrometric raw data to the ProteomeXchange Consortium. Finally, the org.Hs.eg.db (version 3.16.0) package (https://www.bioconductor.org) was used to map gene symbols to their corresponding Entrez gene IDs. Reference gene sets for pathway enrichment analysis were obtained from the Gene Ontology (https://geneontology.org), the Kyoto Encyclopedia of Genes and Genomes (KEGG) (https://www.genome.jp/kegg/), and the Molecular Signatures Database (https://www.gsea-msigdb.org/gsea/msigdb). Differentially expressed proteins were analyzed for over-representation analysis (ORA) using the “enrichGO”, “enrichKEGG”, and “enrichr” functions from the clusterProfiler package (version 4.6.2) [31] to identify enriched GO, KEGG, and Hallmark pathways, respectively. For Gene Set Enrichment Analysis (GSEA), a ranking metric was computed by multiplying the negative logarithm of the p-value by the sign of the log2FC for all proteins, and then sorting the results in descending order. Ranked gene lists were transformed into a named vector format for downstream GSEA implementation with the “gseGO”, “gseKEGG”, and “GSEA” functions. The enriched pathways were visualized using the ggplot2 (version 3.4.4) package. All these pathway analyses were performed in the R environment.

### Lipidomics.

Ex vivo isolated adipocytes (study #1), *in vitro* cultures of adipocyte and precursor cells (study #2), as well as bulk adipose tissue samples (study #3), were handled in the Lipidomics Lab Regensburg, located at the Institute of Clinical Chemistry and Laboratory Medicine of the University Hospital of Regensburg (Germany). Lipid composition was measured by mass spectrometry (MS) for direct flow injection analysis, coupled to liquid (LC) and gas chromatography (GC). An amount of 2 mg wet weight (adipose tissue), 1–10 μg protein (adipose-derived adipocytes), or about 100 μg protein (cultures of primary adipocytes and progenitor cells) were subjected to lipid extraction according to the protocol by Bligh and Dyer [32], with internal standards added to each sample prior to lipid extraction. For flow injection analysis, the lipid extract was vacuum-dried and afterwards dissolved in chloroform/methanol/2-propanol (1:2:4 v/v/v) with 7.5 mM ammonium or in chloroform/methanol (1:1 v/v/v) with 7.5 mM ammonium formate (for measurement of triglycerides). For hydrophilic interaction liquid chromatography (HILIC)-MS/MS, the lipid extract was injected in chloroform. The analysis of lipids was performed by direct flow injection analysis (FIA) using a triple quadrupole mass spectrometer (FIA-MS/MS) and a high-resolution hybrid quadrupole-Orbitrap mass spectrometer (FIA-FTMS). FIA-MS/MS was performed in positive ion mode using the analytical setup and strategy described previously [33]. A fragment ion of *m/z* 184 was used for lysophosphatidylcholines (LPC) [34]. The following neutral losses were applied: Phosphatidylethanolamine (PE) and lysophosphatidylethanolamine (LPE) 141, phosphatidylserine (PS) 185, phosphatidylglycerol (PG) 189 and phosphatidylinositol (PI) 277 [35]. Sphingosine based ceramides (Cer) and hexosylceramides (Hex-Cer) were analyzed using a fragment ion of *m/z* 264 [36]. PE-based plasmalogens (PE P) were analyzed according to the principles described by Zemski-Berry [37]. Cardiolipin was monitored by diglycerol fragment ions [38]. Glycerophospholipid species annotation was based on the assumption of even numbered carbon chains only. Detailed description of the FIA-FTMS method is available in [39]. Triglycerides (TG), diglycerides (DG) and cholesterol esters (CE) were recorded in positive ion mode *m/z* 500–1000 as [M+NH_4_]^+^ at a target resolution of 140,000 (at 200 *m/z*). CE species were corrected for their species-specific response [40]. Phosphatidylcholines (PC), PC ether (PC O) and sphingomyelins (SM) were analyzed in negative ion mode *m/z* 520–960 as [M+HCOO]^−^ at the same resolution setting. Analysis of free cholesterol (FC) was performed after derivatization with acetyl chloride followed by multiplexed acquisition (MSX) of the [M+NH_4_]^+^ of FC (converted to cholesteryl acetate) and the deuterated internal standard (FC[D7]) [40,41]. With regard to HILIC-MS/MS, lipid extracts were subjected to HILIC coupled to MS/MS using a similar setup as described in [38]. A fragment ion of *m/z* 184 was used to quantify PC and SM [33]. Absolute intensities obtained for each lipid and sample were used for analysis. Missing values in the dataset were initially replaced with zeros and subsequently imputed by calculating half of the minimum non-zero value and introducing random noise, as described previously [42]. Relative concentrations were normalized based on the total amount within each lipid class, followed by log_2_ transformation for conducting Student’s t-test between the study and the control group. Differential abundance of lipids was identified based on an absolute log_2_ fold-change (FC)≥0.58 and p-value≤0.05. Quality control analyses were performed in all three substudies, and the resulting plots were visualized using the ggplot2 package (version 3.4.4). Additionally, hierarchical clustering heatmaps based on Euclidean distance were generated utilizing the pheatmap package (version 1.0.12) (https://www.bioconductor.org).

### Mitochondrial function.

For the mitochondrial function test, we used an Agilent Seahorse XF Pro Analyzer. SGBS-derived adipocytes were seeded into XF Pro M cell culture plates (Agilent), differentiated, and subsequently treated with 1% MCM for 72 hours. HAMECs were similarly seeded and treated for 72 hours with MCM or ACM, with the respective controls. Prior to run, cells were washed and incubated for 1 h in XF base medium (#103575) supplemented with 10 mM glucose (#103577), 1 mM sodium pyruvate (#103578), and 2 mM L-glutamine (G7513, Agilent) in a CO_2_-free incubator. During the Seahorse XF Mito Stress Test, Oxygen Consumption Rates (OCR) were monitored at baseline and following sequential injection of oligomycin, carbonyl cyanide-p-trifluoromethoxyphenyl-hydrazone (FCCP), and a mixture of rotenone and antimycin A. Compounds were loaded into the sensor cartridge at 10 μM oligomycin, 20 μM FCCP, and 10 μM rotenone/antimycin A. Following the ~10-fold dilution upon injection into the 180 μL assay medium, these correspond to final concentrations in the assay well of approximately 1 μM oligomycin, 2 μM FCCP, and 1 μM rotenone/antimycin A. Data were acquired and analysed using Wave software (Agilent). The cell number in each well was determined using 3.3 μM Hoechst (# 62249, Thermo) staining together with a Cytation 5 Cell Imaging Multi-Mode Reader (BioTek), and used to normalize flux rates.

### Mitochondrial DNA.

HAMEC were treated for 72 hours with ACM and MCM, alongside their respective controls (n=3/group). DNA was isolated from cell lysates following manufacturer’s instructions (#51304, Qiagen). Mitochondrial DNA (mtDNA) content was assessed by means of a LightCycler 480 II Real-Time PCR System (Roche). Expression of 16S rRNA, Cytochrome b (CYTB), and mitochondrial displacement loop (D-loop) was used as surrogates of mtDNA, while expression of Amyloid Beta Precursor Protein (APP), Beta-2-Microglobulin (B2M), and Ribosomal protein lateral stalk subunit P0 (RPLP0) was used to address genomic DNA (gDNA) content. For each target, 2^(−ΔCT)^ values were calculated, and mtDNA measurements were subsequently normalized taking gDNA values as reference. Forward (Fw) and reverse (Rv) primer sequences are provided below.

16S rRNA: Fw, GGGGCGACCTCGGAGCAGAA; Rv, ATAGCGGCTGCACCATCGGGA

CYTB: Fw, GCCTGCCTGATCCTCCAAAT; Rv, AAGGTAGCGGATGATTCAGCC

D-loop: Fw, CATCTGGTTCCTACTTCAGGG; Rv, CCGTGAGTGGTTAATAGGGTG

APP: Fw, TGTGTGCTCTCCCAGGTCTA; Rv, CAGTTCTGGATGGTCACTGG

B2M: Fw, TGCTGTCTCCATGTTTGATGTATCT; Rv, TCTCTGCTCCCCACCTCTAAGT RPLP0: Fw, TGGTCATCCAGCAGGTGTTCGA; Rv, ACAGACACTGGCAACATTGCGG

### Angiogenesis assay.

Following 72 hours of treatment with 10% ACM, 1% MCM, and respective control on HAMEC cultures, angiogenesis assays were conducted using the ECMatrix^™^ system (ECM625, Millipore). To do so, cells were seeded onto the matrix in 96-well plates according to the manufacturer’s protocol (n=4/group). Tube formation was imaged at 2, 4, 6, and 8 hours using an Invitrogen EVOS^™^ M5000 microscope at 4× magnification. Quantification of angiogenesis was performed on binary images with the Fiji Angiogenesis Analyzer plugin1 (ImageJ) [43], measuring percentage of total segment length and total branching length.

### Data analysis.

To screen out differentially expressed genes (DEG), the series matrix files were downloaded and analysed by NetworkAnalyst 3.0 (http://www.networkanalyst.ca) through Log_2_ transformation normalization. Utilizing Limma, a Log_2_ (fold change)>1 and p-value<0.05 were applied as cut-off criteria. Cluster analysis and integrated bioinformatics analysis were used to visualize the interaction relationships of the DEGs in each dataset. Representative images, including heatmap, the gene cluster in the PCA loading score, and the chord diagram of the datasets, were visualized using NetworkAnalyst 3.0. The KEGG topology enrichment analysis, protein-protein interaction (PPI) topology analysis, and Gene Regulatory Networks TF-gene interactions analyses were also applied. KEGG [44] and GO [45] analyses were performed using overrepresentation analysis methods in the Web-based Gene Set Analysis Toolkit (WebGestalt, http://www.webgestalt.org) and Metascape (http://www.metascape.org). The KEGG topology enrichment and PPI topology analyses were also performed using NetworkAnalyst 3.0. Differentially expressed proteins associated with common GO pathways were analysed to generate alluvial diagrams. GeneRatio values were first converted into a numerical format by computing the ratio of enriched gene counts and background genes. GO pathways were then ranked based on adjusted p-values and GeneRatio values. To construct the alluvial diagram, candidate proteins were first transformed into a long format suitable for visualization. The alluvial diagram was generated using the “geom_sankey” function from the ggsankey package and visualized with ggplot2 (https://www.bioconductor.org), illustrating the flow relationships between proteins and pathways. Additionally, a dot plot was created to display GeneRatio and corresponding pathways with significant adjusted p-values. The final visualization integrates the alluvial diagram with the dot plot, aligning them through coordinate transformations and custom annotations for improved clarity. Statistical analyses were carried out with Mann-Whitney test as stated in figure captions, by using Graphpad Prism 9. In the ADIPOMIT cohort, metabolically “unhealthy” obesity (UO) was defined based on the International Diabetes Federation criteria [46], with the presence of at least one of the following traits: (a) hypertriglyceridemia (HTG), defined as triglyceride [TG]≥1.7 mmol/L (150 mg/dL); (b) dyslipidaemia (DL), defined as high-density lipoprotein cholesterol [HDL] less than 1.03 mmol/L (40 mg/dL) in men, and less than 1.29 mmol/L (50 mg/dL) in women; and (c) hyperglycemia (HG), when fasting plasma glucose≥5.6 mmol/L (100 mg/dL). Participants with one or more of these metabolic alterations were categorized into the UO group, while participants with none of the above-mentioned abnormalities were categorized into the “healthy” obesity (HO) group [47]. For this study, raised blood pressures, as well as the presence of central obesity, defined as waist circumference≥94 cm for men, and ≥80 cm for women, were neglected, as hypertension and central obesity is assumed when BMI≥30 kg/m^2^. For conducting Stepwise Regression Analysis, normalized Log_2_-transformed gene expression data and clinical covariates (i.e., age, sex, and BMI) were used. The outcome variable was group classification (UO vs HO), while predictors consisted of gene expression values together with covariates. Stepwise regression was performed in R using the stepAIC function from the MASS package (v7.3) (https://www.stats.ox.ac.uk/pub/MASS4/), starting from the full model and applying bidirectional selection based on the Akaike Information Criterion (AIC). The performance of the final logistic regression model was evaluated using receiver operating characteristic (ROC) curve analysis with the pROC package (v1.19) [48]. Finally, variable importance scores were calculated using multiple machine learning approaches implemented in R to assess the relative contribution of each predictor. Models included logistic regression from the caret package (v7.0) [49], random forest from the randomForest package (v4.7) (https://doi.org/10.1023/A:1010933404324), decision tree from the rpart package (v4.1) (10.32614/CRAN.package.rpart), support vector machine from the caret package, extreme gradient boosting (XGBoost) from the caret package, and LightGBM from the lightgbm package (v4.6) (10.32614/CRAN.package.lightgbm). Variable importance was quantified using Mean Decrease in Gini for the random forest model, the total decrease in Gini impurity for the decision tree model, and the default gain feature-importance metric for both the XGBoost and LightGBM models. Predictors were thus ranked by importance, and the top 15 features were visualized using ggplot2 package (v4.0) (https://ggplot2.tidyverse.org). The same models were re-fit in the KOBS cohort, consisting of the subcutaneous adipose tissue bulk RNA-seq of 241 bariatric patients (BMI>30 kg/m^2^), where 22 individuals showed HO, and 219 were segregated as UO participants, as defined above.

## RESULTS

### Adipocytes in obesity show suppressed mitochondrial programs and altered lipid handling.

To resolve cell-type-specific alterations associated with obesity, we performed the segregation and multi-omic profiling of adipocytes and cultured EC sourced from the adipose tissue of an initial sample of 9 participants in the lean BMI range (BMI<25 kg/m^2^) and 8 participants living with obesity (BMI>30 kg/m^2^) (**Table S1**). We focused first on obesity-associated defects intrinsic to adipocytes. Untargeted LC–MS proteomics of ex vivo isolated adipocytes from 3 lean-range and 5 donors with obesity identified ~1,400 protein groups retained for downstream analyses (**Figures S1a-b**). PCA (Figure 1a) and hierarchical clustering (**Figure S1c**) showed clear separation by BMI group. Using a significance cut-off of nominal p-value<0.05, a total of 459 proteins were identified as differentially abundant proteins (DAPs) by comparing proteomes of adipocytes from participants with obesity to those from lean participants, including 137 increased and 322 decreased proteins (Figure 1b). Enrichment analyses indicated that mitochondrial energy metabolism is the dominant axis suppressed in obese adipocytes. Over-representation analysis (ORA) and gene ontology (GO) highlighted downregulation of cellular and aerobic respiration and related energy-derivation processes, with cellular component enrichment mapping respiratory chain and mitochondria-related complex structures (Figure 1c and **Table S7**). Consistently, Gene Set Enrichment Analysis (GSEA) using the Kyoto Encyclopedia of Genes and Genomes (KEGG) showed negative enrichment of oxidative phosphorylation (OXPHOS), citrate (TCA) cycle, and valine/leucine/isoleucine degradation (adjusted p-value=4.73E-9), together with coordinated reductions in other mitochondrial substrate-handling pathways (Figure 1d). Reactome analyses also indicated increased trafficking-related processes, including intra-Golgi and retrograde Golgi-to-ER transport (**Figure S1d**), supported by the enrichment of COPI-coatomer components (COPA, COPB1, COPB2, COPG1, etc.) together with ADP-ribosylation factor 4 (ARF4), dynein/dynactin, and tubulin subunits. Lipidomic profiling of isolated adipocytes was dominated (>99% of total lipid) by triglycerides (TG), necessitating relative rather than absolute quantification across lipid classes (Figure 1e). We identified 56 lipid species differing between groups (nominal p-value<0.05). Most TG species were preferentially depleted in adipocytes from subjects with obesity, whereas some sphingomyelin (SM), phosphatidylethanolamine (PE), ether-linked phosphatidylcholine (PC O), phosphatidylcholin (PC), and diglyceride (DG) species were enriched (Figure 1e). Notably, TG with shorter fatty acid chains (≤46 carbons total) and higher saturation (≤3 double bonds) were less abundant in adipocytes from participants with obesity as compared to lean-range (Figure 1f). This compositional shift aligned with the proteomics data showing reduced abundance of enzymes supporting de novo fatty acid biosynthesis (e.g., PERC, FASN, ACACA) and mitochondrial β-oxidation (HADHA, HADHB, etc.) (Figure 1b), consistent with impaired lipid plasticity and oxidative capacity. On the other hand, metabolomics (**Figures S2a-c**) identified 30 metabolites differing (nominal p-value<0.05) between BMI groups (16 increased, 14 decreased). Metabolites increased in adipocytes from subjects with obesity included L-glutamic acid, glutathione, thiamine pyrophosphate, myo-inositol, N-acetylneuraminate, and L-carnitine, while several fatty acids were reduced (**Figures S2d**). Also in line with our proteomic results, fatty acid biosynthesis (6/29, p=4.1E-4) emerged as the most prominently altered process (**Figure S2e**). Together, these ex vivo data define an obesity-associated adipocyte state characterized by suppressed mitochondrial energy metabolism and remodelled TG distribution consistent with altered lipid handling.

### EC in obesity exhibit reduced cell-cycle signalling and increased mitochondrial protein abundance.

We next mapped obesity-associated changes in the adipose microvasculature by profiling cultures of adipose-derived EC from 7 lean-range participants (BMI<25 kg/m^2^) and 5 subjects with obesity (BMI>30 kg/m^2^), using flow cytometry/cell sorting and untargeted proteomics (**Figures S3a-b**). PCA (Figure 2a) and hierarchical clustering (**Figure S3c**) showed clear segregation by BMI group. We identified 439 DAP (nominal p-value<0.05), including 289 decreased and 150 increased proteins in EC from participants with obesity (Figure 2b). Hallmark GSEA revealed enrichment of mitochondrial and lipid-handling programs, including OXPHOS (NES=2.36, p=1.23E-9), adipogenesis (NES=2.15, p=3.56E-7), and fatty acid metabolism (NES=1.48, p=4.51E-2), accompanied by downregulation of proliferation-associated pathways (i.e., E2F targets, G2M checkpoint, and Myc targets) and mTORC1 signalling (Figure 2c). Reactome analyses supported enrichment of insulin receptor signalling and aerobic respiration/electron transport (**Figure S3d**). Of note, several mitochondrial proteins apparently suppressed in adipocytes from participants with obesity were regulated in the opposite direction in EC (Figure 2d), namely TCA cycle-related proteins (e.g., KYAT3, DLD, CYC1, PDHB, DLST, SUCLG2, ACAA2, ACAT1, NDUFAB1, MDH2, PPA2) and fatty acid β-oxidation proteins overlapping with branched-chain amino acid (BCAA) catabolism (DECR1, HADHA, HADHB) (Figure 2e). These data indicate a cell-type specific phenotype divergence in obesity: mitochondrial programs are suppressed in adipocytes yet enhanced at the protein level in adipose-resident EC, alongside reduced cell-cycle signalling.

### Macrophage-induced inflammation partially reproduces obesity-related adipocyte alteration.

To test whether inflammation is sufficient to induce the adipocyte phenotype observed in subjects with obesity, we exposed cultures of adipocytes to macrophage-conditioned media (MCM). As a technical prerequisite, we first verified expected proteome and lipidome remodelling during adipocyte differentiation in vitro (**Figures S4a-c**), confirming progressive enrichment of canonical adipocyte features encompassing significant lipidome modulation (**Figures S5a-f** and **Table S8**). We then modelled obesity-associated inflammatory stress by exposing differentiated adipocytes to MCM (**Figures S4d-f**). PCA on proteomic profiling data showed clear separation between control and MCM-treated adipocytes (Figure 3a). In fact, MCM altered 2,163 proteins (nominal p-value<0.05), including 1,218 decreased and 945 increased (Figure 3b). ORA (GO) identified functional enrichment of mitochondrial metabolism as the dominant suppressed axis, including depletion of mitochondrial matrix, inner membrane, and respiratory complex components (Figure 3c and **Table S9**). KEGG GSEA (Figure 3d) further demonstrated coordinated downregulation of OXPHOS (NES=−2.47), BCAA degradation (NES=−2.36), carbon and propanoate metabolism, thermogenesis, and citrate cycle (adjusted p-value=2.24E-9). Functionally, MCM reduced oxygen consumption rate (OCR) in SGBS-derived adipocytes (**Figure S4g**), driven by impaired basal respiration (adjusted p-value=0.0054) and reduced ATP-linked respiration (adjusted p-value=0.0225). Noteworthy, MCM also induced TG remodelling partially consistent with obesity-related alterations in adipocyte lipidomes: decreased shorter, more saturated TG species, while longer, more unsaturated TG species were consistently increased (Figures 3e–f). Thus, macrophage-derived inflammatory signals are sufficient to reproduce key obesity-associated features in adipocytes, including suppression of mitochondrial pathways and coordinated lipid remodelling.

### Immune and adipocyte-derived cues differentially shape EC adaptations.

To dissect upstream signals contributing to the EC phenotype observed in obesity, we studied human adipose microvascular endothelial cells (HAMEC) under culture conditions modelling a pro-inflammatory adipose milieu (Figure 4a). Exposure to MCM induced impaired respiration (Figure 4b), broad pathway disruption marked by inflammatory responses (e.g., interferon alpha/gamma response, TNFα signalling via NFκB, inflammatory response), and suppression of cell-cycle and bioenergetic pathways (**Figures S6a-g** and **Table S10**), together with reduced translation and altered extracellular matrix organization (Figure 4c). Functionally, MCM-treated EC displayed higher sprouting initiation, anchorage junctions, branching, and segment length (Figure 4d), consistent with endothelial remodelling compatible with endothelial–mesenchymal transition [50,51]. As the downregulation of mitochondrial proteins induced by MCM was in opposition to the increased mitochondrial protein abundance observed in adipose-resident EC obtained from participants with obesity, we next tested whether adipocyte-derived cues contribute to the mitochondrial component of EC. Adipocyte-conditioned media (ACM) from MCM-activated adipocyte cultures increased non-mitochondrial oxygen consumption, maximal OCR, ATP-linked respiration, and spare respiratory capacity in EC (Figure 4e). ACM induced 459 increased and 624 decreased proteins (nominal p-value<0.05) in HAMEC (**Figures S7a-e**), including mitochondrial protein fluctuations affecting biogenesis, organization, translation, ATP synthesis, and electron transfer (Figures 4f and **S7f**, and **Table S11**). ACM (but not MCM) also increased mitochondrial DNA content (Figure 4g), and coincided with repression of glycolysis (**Figure S7g**) and glycolytic proteins (**Figure S7e**), consistent with a shift toward mitochondrial energy production. Through the scrutiny of OXPHOS and Myc target-annotated proteins, we further confirmed increased OXPHOS and decreased cell-cycle signalling in EC from participants with obesity (Figure 4h). While MCM treatments led to the downregulation of Myc target genes, mirroring the aforementioned observations, ACM functional measurements provided insights into the enrichment of mitochondrial proteins, supporting the dual impact of inflamed macrophages and adipocytes on EC dysfunction within the context of obesity. Together, these findings support a dual-source model of EC dysfunction in obesity: immune-cell inflammation shapes quiescence/angiogenic remodelling [52], whereas adipocyte-to-EC communication may preferentially contribute to altered mitochondrial activity and respiration [53].

### Surgery-induced weight loss remodels bulk adipose tissue toward mitochondrial activation and lipid plasticity.

We next asked whether obesity-linked adipose programs recover (i.e., shift in the opposite direction) after weight loss. We profiled paired abdominal adipose tissue from eighteen participants with severe obesity before and after ~2 years of surgery-induced weight loss (**Table S2**). Participants transitioned from class III obesity into the overweight range, with BMI decreasing from 42.8±4.9 to 28.6±5.5 kg/m^2^ (p=9E-11) and fat mass from 55.7±7.0% to 39.5±7.3% (p=2.6E-10). As expected [54], HDL cholesterol increased from 54.7±14.1 to 74.9±24.1 mg/dl (p=9.9E-5). Untargeted in-depth LC–MS proteomics of the adipose tissue yielded 6,694 protein groups. After filtering (**Figures S8a-b**), 5,842 proteins were retained. Hierarchical clustering (**Figure S8c**) and PCA (Figure 5a) segregated samples by weight loss status. Comparing post-weight loss to baseline yielded 2,012 DAPs (nominal p-value<0.05), 1,154 increased and 858 decreased (Figure 5b). ORA (GO) showed strong enrichment of mitochondrial biological processes and components (Figure 5c and **Table S12**), including mitochondrial matrix and protein-containing complexes. KEGG GSEA indicated robust upregulation of mitochondrial and substrate-handling pathways after weight loss (Figure 5d), including valine/leucine/isoleucine degradation (NES=2.36), TCA cycle (NES=2.32), OXPHOS (NES=2.3), pyruvate metabolism (NES=2.17), and fatty acid metabolism (NES=2.11). In parallel, inflammation-related signalling significantly decreased, including complement/coagulation cascades, C-type lectin and Toll-like receptor signalling (Figure 5d). Reactome GSEA also suggested normalization of COPI-mediated and Golgi–ER transport pathways (**Figure S8d**). On the other hand, lipidomics identified 122 lipid species altered by weight loss (nominal p-value<0.05). Several membrane glycerophospholipid and sphingolipid classes increased, while free cholesterol and multiple DG species decreased (Figure 5e). TG remodelling was the most prominent shift: TG species with shorter fatty acids (≤47 carbons) and relatively higher saturation (≤3 double bonds) increased, whereas TG species with longer chains (≥50 carbons) decreased (Figure 5f). Integrating proteomics with lipidomics demonstrated concordant recovery of de novo lipogenic enzymes (ACLY, ACACA, FASN, etc.) and mitochondrial β-oxidation/BCAA metabolic process proteins (HADHA, HADHB, ACADM, etc.) alongside the shift in TG species, which was opposite in direction to observations in cultures of inflamed adipocyte and adipocytes isolated from subjects with obesity (**Figures S9a-c**). As this series of analysis profiled bulk adipose tissue rather than isolated fat cells, the post-weight loss signature likely reflects both adipocyte-intrinsic remodelling and changes in cellular composition, together with alterations of the inflammatory activity within adipose tissue after malabsorptive surgery.

### A robust adipocyte gene set emerges by intersecting obesity, inflammation, and weight loss-reversal across platforms.

To pinpoint adipocyte determinants that consistently track adipocyte inflammation driving adipose dysfunction and its reversal after weight loss, we intersected proteomic findings across (i) ex vivo isolated adipocytes in obesity, (ii) in vitro MCM-treated adipocytes, and (iii) bulk adipose tissue before-after weight loss. Proteins decreased in adipocytes from participants with obesity and/or MCM-treated adipocytes but increased after weight loss (3-way-or-2-of-3 criterion) comprised 463 candidates (Figure 6a). GO enrichment of this intersection highlighted mitochondrial processes and fatty acid metabolism remodelling, with mitochondrial cellular components dominating the signal (Figure 6b). To validate that these protein-level candidates generalize beyond our datasets, and to benchmark directionality also at the transcript level, we cross-referenced the only 3-way criterion 177 proteins against two independent transcriptomic resources (Figure 6c). In reference [55], adipose biopsies from 50 patients with obesity were profiled at baseline and 2 years after surgery-induced weight loss. In reference [56], O’Hara *et al*. profiled transcript changes in differentiated SGBS adipocytes [57] exposed to 14% MCM. Of the 177 protein candidates, 86 protein-coding transcripts recapitulated our directionality, i.e., upregulation correlated to adipose tissue shrinkage after weight loss (Figure 6d), as well as downregulation in adipocytes exposed to inflammation (Figure 6e), presenting a core set enriched for mitochondrial function and BCAA metabolism. Conversely, proteins decreased after weight loss and/or increased in adipocytes from participants with obesity and/or in MCM-treated adipocytes (3-way-or-2-of-3 criterion) comprised 156 candidates (Figure 7a). GO enrichment emphasized intracellular vesicle transport and cell–substrate junction pathways, with corresponding enrichment of vesicle coat/lumen, COPI-associated structures and adhesion-related binding functions (Figure 7b). Filtering these 156 candidates against transcripts downregulated with weight loss in bulk fat [55] and induced by MCM in adipocytes [56] yielded a refined list of 41 genes (Figure 7c), summarized by alluvial diagrams linking protein-coding genes to key pathways (Figure 7d–e). Notable components encode constituents for the regulation of cell–substrate adhesion, cell adhesion and molecule binding, extracellular exosome, and vesicle-mediated transport. Together, the 86 weight loss-reversible mitochondrial/BCAA-linked genes, and the 41 vesicle/adhesion-linked genes define a 127-gene signature that, in adipocytes/bulk fat, is consistently altered in obesity and inflammatory stress, and shifts in the opposite direction with weight loss across different platforms. A summary of proteomics data and the study pipeline is provided in **Figure S10a**.

### The adipocyte signature tracks metabolic vulnerability in independent cohorts and yields a 38-gene predictor of metabolic obesity.

To connect the adipocyte signature to physiology at population scale, we re-analysed subcutaneous adipose RNA-seq data from three cohorts (**Figure S10b**): 335 unrelated male participants (median age 54 ± 5 years, BMI=26.8 ± 3.7 kg/m^2^) from the Finnish METabolic Syndrome In Men (METSIM) cohort [21,22] (**Table S3**); 49 BMI-discordant monozygotic twin pairs (Finnish Twin Cohort) [23]; and 451 adults with obesity (31% men; median age 46 ± 10 years, BMI=46.8 ± 7.9 kg/m^2^) from the multi-centered Spanish ADIPOMIT cohort [24] (**Table S4**). In METSIM, we tested associations with BMI, HDL cholesterol, fasting TG, and blood glucose after adjusting for BMI, age, and type 2 diabetes (Figure 8a). This benchmarking study gave us an overview of our previous meta-associations in a sample representing a male population prone to cardiometabolic complications, exceeding those in females [58]. In BMI-discordant twins, 95 of the 127 candidates (74.8%) were differentially expressed (FDR adjusted p-value<0.05) in the heavier co-twin (Figure 8b), supporting their link to adiposity independent of the genetic background. In ADIPOMIT, meta-analysis of gene–trait associations (Figure 8c) showed that only 60 candidates (47%) correlated with BMI (nominal p-value<0.05), whereas in METSIM 115 candidates (91%) did so. We next assessed whether our 127-gene signature stratifies metabolic vulnerability among people living with obesity in ADIPOMIT. Individuals with one or more metabolic abnormalities (i.e., dyslipidaemia, hypertriglyceridemia, and/or hyperglycaemia) were classified as “unhealthy” obese (UO), and those with none as “healthy” obese (HO). Of note, these terms are employed as abbreviations; they reflect selected metabolic abnormalities but do not necessarily define a “healthy” or “unhealthy” state. Using the single-sample Gene Set Enrichment Analysis (ssGSEA) algorithm [59], a gene-set score per sample was associated with the presence of comorbidity (Figure 8d) and distinguished HO from UO with an AUROC of 0.7 (0.63–0.77) (Figure 8e). Stepwise regression including age, sex, and Log_2_ BMI as covariates selected 38 genes (Figure 8f) that substantially improved discrimination (Figures 8g–h), yielding an AUROC of 0.92 (0.89–0.95). Thus, the adipocyte signature not only tracks obesity and inflammation across datasets but also captures clinically relevant metabolic vulnerability among people living with obesity.

### Integrating adipocyte and EC gene candidates yields a broader genomic signature of cardiometabolic vulnerability.

We next extended the framework to include EC-associated protein patterns linked to obesity-related meta-inflammation (**Figures S11a-b**). Integrative analysis of adipose-derived EC and HAMEC cultures yielded 237 proteins with patterns negatively correlating with obesity (Figure 9a) and 91 proteins defining an inflamed EC phenotype (Figure 9b). As above, we applied here the relaxed 3-way-or-2-of-3 criterion, due to the low number of proteins meeting strict 3-way overlap. Using funnel-diagram pipelines, we shortlisted EC-specific features that replicate weight loss-associated expression patterns in bulk adipose transcriptomes [23,55,60], generating a set of 34 EC genes (19+15) mostly associated with protein translation and endo/lysosome function (**Figures S12a-c**), consistent with dysregulated protein synthesis and compromised lysosomal degradation in obesity/inflammation. However, when queried in the ADIPOMIT cohort, our EC-type-resolved gene signature showed limited correlation with metabolic comorbidities (Figure 9c and **Figure S12d**). Next, forward/backward stepwise AIC selection across 159 protein candidates (125 adipocyte-specific, and 32 EC-derived genes, plus 2 common proteins, as shown in **Figure S12e**) identified in this study yielded a new model including 36 adipocyte- and 10 EC-derived genes (Figures 9d); a refined, final 46-gene signature capturing metabolic distress in subjects with obesity with an AUROC of 0.95 (0.92–0.97) (Figure 9e). Notably, the combined adipocyte/EC model showed a statistically significant improvement over the adipocyte-only model (Z=2.0001, p-value=0.04549). Indeed, across six machine learning algorithms distinguishing UO from HO, both adipocyte (ACADSB, BCAT1, BCKDHB, CBR4, CD163, CKB, CPPED1, DLST, FASN, IVD, LCP1, LMNB1, LPXN, MIF, NCEH1, NDUFA5, PDHX, PECR, PTPRJ, SERPINB8, and SHMT1) and EC genes (CALU, CTSS, and RPS4X), together with BMI (Log_2_ BMI), consistently ranked among the top contributors for metabolic obesity in the ADIPOMIT cohort (**Figure S13a**). To replicate our findings on an independent sample of individuals with obesity, we utilized the Kuopio OBesity Surgery (KOBS) dataset [4]. This confirmatory study of bulk subcutaneous adipose tissue RNA-seq conducted in 241 bariatric patients, where 22 individuals showed HO, and 219 were segregated in UO participants (**Table S5**), validated our 46-gene signature (Figure 9f) with an AUROC of 0.93 (0.88–0.99) (Figure 9g), while machine learning algorithms (**Figure S13b**) further confirmed the relevance of at least 15 adipocyte- and EC-related gene candidates (including BCAT1, CALU, CDK6, COQ9, CPPED1, CTSS, DLD, NDUFA5, NRP2, PCCA, PECR, SEC16A, SHMT1, UCHL3, and UQCRC2) in defining cardiometabolic vulnerability in people with obesity (**Figure S13c**).

## DISCUSSION

Obesity is accompanied by adipose tissue dysfunction linking excessive adiposity and inflammation to cardiometabolic disease, yet the programs responsible are difficult to attribute to specific cell types and distinguish in experimental settings from protective changes accompanying weight loss. Here, we used a multi-layered design to (i) define obesity-associated, cell-type-resolved molecular phenotypes in adipocytes and adipose-resident EC; (ii) mimic an obesity-like inflammatory milieu in vitro to probe macrophage-adipocyte-endothelial signalling by means of macrophage and adipocyte-conditioned media; and (iii) determine which obesity/inflammation-associated proteins and lipids show evidence of recovery in bulk adipose tissue after weight loss. The resulting protein signatures were then intersected with transcriptomic datasets of weight loss [55] and inflamed adipocytes [56], and evaluated for physiological relevance across several human cohorts (METSIM [22], BMI-discordant twins [23], ADIPOMIT [24], and KOBS [4]). It yielded a refined, non-immune cell-derived 46-gene signature of metabolic vulnerability in people with obesity. Together, these findings support a model in which obesity-associated adipocyte and EC dysfunction, albeit initially driven by nutrient overload, is largely inflammation-responsive, and shows evidence of reversibility with weight loss. A diagram summarizing our research and main conclusions is presented in Figure 10.

Our integrative approach confirmed cell-type specific mitochondrial malfunction in adipocytes as a prominent feature of obesity, marked by a significant impairment of adipocyte respiration and normalized to a significant extent upon weight loss [61]. Given the bulk tissue nature of the measurements in vivo, our observations may reflect a combination of reduced nutrient overload, adipose tissue remodelling, adipocyte shrinkage, and reversal of the mitochondrial defects, rather than uniform adipocyte cell-intrinsic recovery alone. However, the present data support the notion that adipose tissue alterations predominantly reflect pathological cues occurring within adipocytes [62]. Replication in adipocyte cultures when challenged with macrophage-derived cytokines further supports a pivotal role of immune inflammation and macrophage-to-adipocyte communication [63,64]. Conversely, intersection of proteins exhibiting an inverse pattern (i.e., decreased in adipose tissue upon weight loss and more abundant in obese/inflamed adipocytes) highlighted vesicle transport and cell-substrate junctions. The former aligns with the increased secretion of obese, inflamed adipocytes, reflecting altered endocrine function and stress adaptation [65,66]. Notably, major coatomer components critical for endoplasmic reticulum-Golgi vesicle trafficking were identified (CLTC, COTL1, COPG1, COPB2, etc.), further corroborating a connection between obesity and the release of extracellular vesicles [67], which is alleviated after weight loss [68]. The overrepresentation of cell-substrate components of junctional complexes is also consistent with reports on mechanophysical interactions with the extracellular matrix (ECM), contributing to adipose tissue remodelling in obesity [69,70]. For instance, dynamic mediators of adipose cell adhesion, such as adipocyte adhesion molecule (e.g., ACAM), can control the development of obesity [71], while modification of focal adhesions deeply impacts the adipogenic transformation of mesenchymal stem cells [72,73], and deletion of integrin-mediated cell adhesion proteins such as integrin-β1 (ITGB1) [74] and kindlin-2 (FERMT2) [75] alters adipocyte function, accompanying excessive adiposity.

By intersecting our findings with publicly available transcriptomes characterising adipose tissue upon weight loss [55] and adipocyte inflammation [56], we shortlisted 127 genes consistently altered in “obese” adipocytes, on both transcript and protein levels. The clinical relevance of these biomarkers was further supported by their association with metabolic distress, including dyslipidaemia, hypertriglyceridemia, and hyperglycaemia across two independent human cohorts (METSIM [22] and ADIPOMIT [24]) and a cross-sectional study conducted in BMI-discordant twins [23]. Refinement using stepwise regression analysis yielded a core set of 38 genes that, when combined, substantially enhanced diagnostic capacity for assessing gene-trait associations and the risk of metabolic disruption in subjects with obesity. This set of gene products represents in adipocytes a promising target, not only for extending our mechanistic understanding of the pathophysiology of obesity, but also for guiding the development of novel diagnostic tools and therapeutic interventions aimed at mitigating the comorbidities of obesity.

In this context, significant alterations in lipid signatures have proven important in tackling adipose tissue function and energy handling [76]. In our complementary approaches, a reduction in TG with comparatively short, saturated fatty acid chains was observed in adipocytes under obese and inflammatory conditions, while being enriched in adipose tissue upon weight loss. In human adipocytes, the relevant fatty acid chains are largely derived from *de novo* lipogenesis (DNL) [77]. Our finding aligns with previous observations [76], and corroborates diminished DNL in obese/inflamed adipocytes, also reflected in the depletion of major DNL enzymes in our proteomic data. Moreover, a reduction in the relative proportion of long-chain, polyunsaturated fatty acid (PUFA)-containing TG species upon weight loss coincides with an increase upon pro-inflammatory treatments on primary adipocytes. This replicates studies reporting elevated PUFA in the adipose tissue of subjects with obesity and type 2 diabetes patients [76,78], as well as mice on high fat diets [79]. The enrichment of PUFA-containing TG species raises questions on how and why distinct TG are generated in adipocytes, and whether they play relevant roles in the development of metabolic disturbances. Interestingly, a PUFA subset (derived from ω−6 PUFA) is considered pro-inflammatory, while others (obtained from both ω−3 and ω−6 PUFA) contribute to the resolution of innate immune activation in adipose tissue [68,80].

On the other hand, the proteomic profiling of cultures of adipose-derived EC obtained from individuals with obesity hinted at a quiescent phenotype, characterized by low abundance of cell cycle-related proteins and consistent with the reduced vasculature and angiogenic capacity of hyperplastic fat pads [12,81]. Paradoxically, enhanced mitochondrial activity and fatty acid oxidation were also observed in “obese” EC. This may be due to the enhanced availability of fatty acids, known to leak out from hypertrophic, insulin-resistant adipocytes [82,83], or might indicate adaptive responses to chronic nutrient excess, adipose tissue expansion and hypoxic states. Of note, respiratory chain components were also elevated in “obese” EC, putatively yielding excessive ROS production. The resulting oxidative stress can induce EC dysfunction, leading to the sustained activation and increased adhesion of immune cells, while contributing to the leakiness of vascular walls [84,85], perpetuating adipose tissue inflammation. As EC mostly rely on glycolysis for energy production [86,87], the upregulation of mitochondrial components likely underscores a change in cellular energetics reflecting quiescent EC, which employ FA oxidation and the TCA cycle to support cellular redox balance against oxidative damage [88].

In this regard, while the treatment of EC cultures with MCM recapitulated the reduction of cell cycle-related proteins accompanying inflammatory activation, the overrepresentation of proteins related to mitochondrial function did not, suggesting the secretome of “obese” adipocytes to be required for full induction of the obese phenotype in adipose-resident EC. Indeed, exposure of EC to media conditioned with inflamed adipocytes (ACM) resulted in increased abundance of several proteins related to mitochondrial respiration and boosted oxygen consumption, together with pathway alterations also observed in “obese” EC and MCM-treated HAMEC. Collectively, these data support the view that pro-inflammatory signals from activated macrophages are crucial for adipose tissue dysfunction, both (i) altering adipocyte lipid and energy metabolism, vesicle transport and adhesion properties, while (ii) reprogramming EC energy metabolism through the influence of inflamed adipocytes with far reaching consequences in bulk adipose tissue and whole-body metabolism [89,90].

### Study strengths and limitations.

This study encompasses several strengths. First, we employed adipocytes and primary cultures of EC isolated from human fat biopsies, preadipocytes and HAMEC cultured in vitro, and in vivo analyses of bulk adipose tissue samples, as well as their cross-section to identify protein and lipid signatures skewed by obesity and inflammation. Second, the inclusion of adipocytes and EC broadens the examination of the impact of excess weight, inflammation, weight loss, and other obesity-related outcomes across two of the most relevant adipose-resident, non-immune cell types. Third, we solidified our findings by filtering protein patterns against transcriptomic data, yielding highly credible lists of genes/proteins dysregulated in obesity. In addition, by using four independent human cohorts, we carefully shortlisted our cell signatures against a comprehensive range of obesity-related outcomes to disentangle the most significant cell-resolved molecular phenotypes of metabolic vulnerability in subjects with obesity, assessing not only the association with adiposity, but also their capacity to predict metabolic outcomes with very convincing results (AUROC curves>0.9).

There are also limitations to consider. These include the descriptive nature of this work, as well as the modest sample size employed for our initial omics analyses, which substantially limited statistical power. In addition, we lacked data on some potential confounding variables, and our study cohorts focus on white European individuals between the ages of 40 to 70. Therefore, caution should be applied when extrapolating our findings to other populations. However, in many other ways, the multiple transcriptomic cohorts queried, when levelled by means of cell-type-resolved and bulk fat protein profiles, hit cross-validation at both protein, lipid and mRNA levels, adding confidence in the robustness of our main conclusions. A further limitation is that different adipose depots were employed in our distinct sub-studies, i.e., adipose samples obtained laparoscopically from the intra-abdominal adipose tissue of the greater omentum (visceral) for cell type-specific analyses, and bulk subcutaneous adipose tissue in the bariatric surgery arm and for cross-sectional confirmation. We acknowledge the well-established differences in the phenotype and cellular composition of visceral and subcutaneous depots of adipose tissue [91]. Therefore, molecular patterns in bulk adipose tissue may reflect depot-specific biology, changes in cellular composition, or differences between cell-resolved and bulk tissue measurements, rather than recovery of the identical adipocyte-intrinsic program. Likewise, striking similarities in the molecular profiles presented here support the notion that the principal mechanisms underlying obesity-associated adipose tissue dysfunction are largely shared between both depots. Finally, the analysis of lipid species in adipose-derived adipocytes was limited to relative quantification due to the technical challenge posed by extremely high triglyceride contents. Therefore, our results do not allow conclusions on absolute depletion or enrichment of lipid species, but only hit on shifts in their relative proportions.

### Conclusions.

Together with inflammation-responsive adipocyte mitochondrial and lipid-handling dysfunction, our cell-type-informed framework probes macrophage and adipocyte-to-endothelial communication in obesity, and delineates cross-context cellular programs associated with weight loss. By integrating adipose-resident, non-immune cell-context-resolved signatures with multiple transcriptomic data obtained in bulk fat, we derived a compact gene set with strong discriminative capacity for cardiometabolic vulnerability in people with obesity. Overall, although the descriptive, correlative nature of our data cannot offer causal insight, the current study constitutes a resource to guide future experimental investigations of protein and lipid targets with an impact on the commitment of bona fide adipose-resident cells and adipose welfare with potential cardiometabolic implications.

## Supplementary Material

Supplementary Files

This is a list of supplementary files associated with this preprint. Click to download.

- ChaurasiyaetalSupplFilesCardiovascDiabetol.pdf
- ChaurasiyaetalSupplementalDataCardiovascDiabetol.xlsx

## Figures and Tables

**Figure 1.**
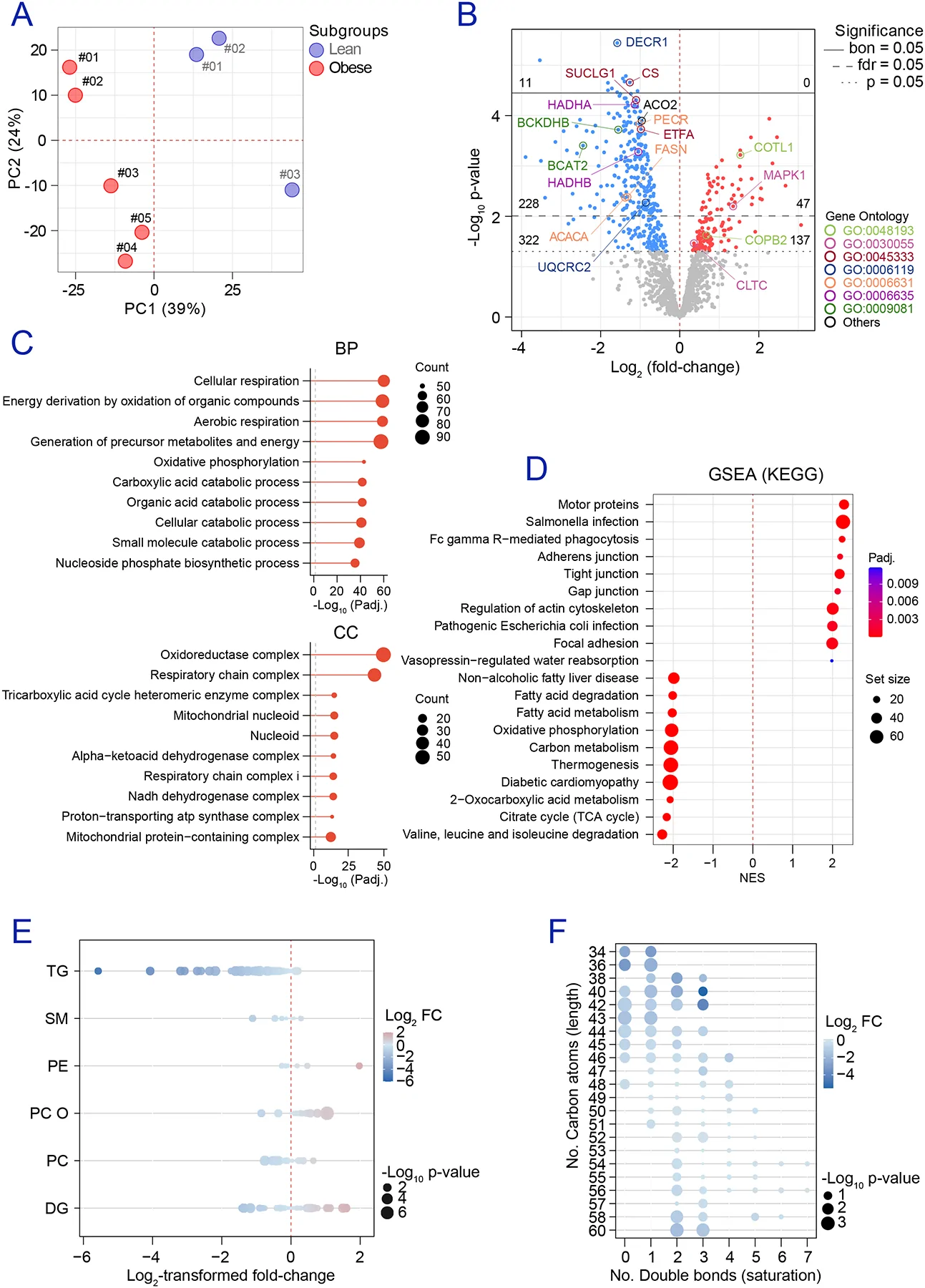
Obesity influences adipocyte protein and lipid-signatures. **(a)** PCA shows the clustering of adipose cells obtained in gallstone surgery patients with (BMI>30 kg/m^2^) and without (BMI<25 kg/m^2^) obesity. **(b)** The scatter dot plot shows DAPs up (red) and down-regulated (blue) in “obese” (n=5) vs “lean” (n=3) adipocytes. Statistical significance was assessed by employing two-tailed Bayesian moderated t-tests and assuming equal variance in unpaired comparisons. Dots in grey show proteins meeting our exclusion criteria (nominal p-value≥0.05). Coloured labels indicate protein landmarks of GO pathways significantly regulated after weight loss (WL), i.e., golgi-vesicle transport (GO:0048193), cell-substrate junction (GO:0030055), cellular respiration (GO:0045333), oxidative phosphorylation (GO:0006119), fatty acid metabolism (GO:0006631), fatty acid beta-oxidation (GO:0006635), and branched-chain amino acid metabolism (GO:0009081). **(c)** ORA in a GO computational framework. Additional details are provided in **Table S7**. **(d)** GSEA (KEGG) annotations revealed, among others, the downregulation of metabolic processes related to mitochondrial performance and lipid handling in subjects with obesity. **(e)** Lipidomes conducted in “obese” (n=8) vs “lean” (n=9) adipocytes show variations on the abundance of triglycerides (TG), sphingomyelins (SM), phosphatidylethanolamines (PE), phosphatidylcholine-ethers (PC O) and phosphatidylcholines (PC), and diglycerides (DG). Other lipid families were dismissed due to the very low amounts detected. Bubble plot in **(f)** shows variations in TG species, segregated according to their saturation (n° double bonds) and length (n° carbon atoms). Differential abundance of lipids was considered significant based on an absolute log_2_ FC value≥0.58 and nominal p-value<0.05. Statistical significance was conducted by means of two-tailed Student’s t-tests. Source data for Figures 1c and 1d is provided as a **Source data file**.

**Figure 2.**
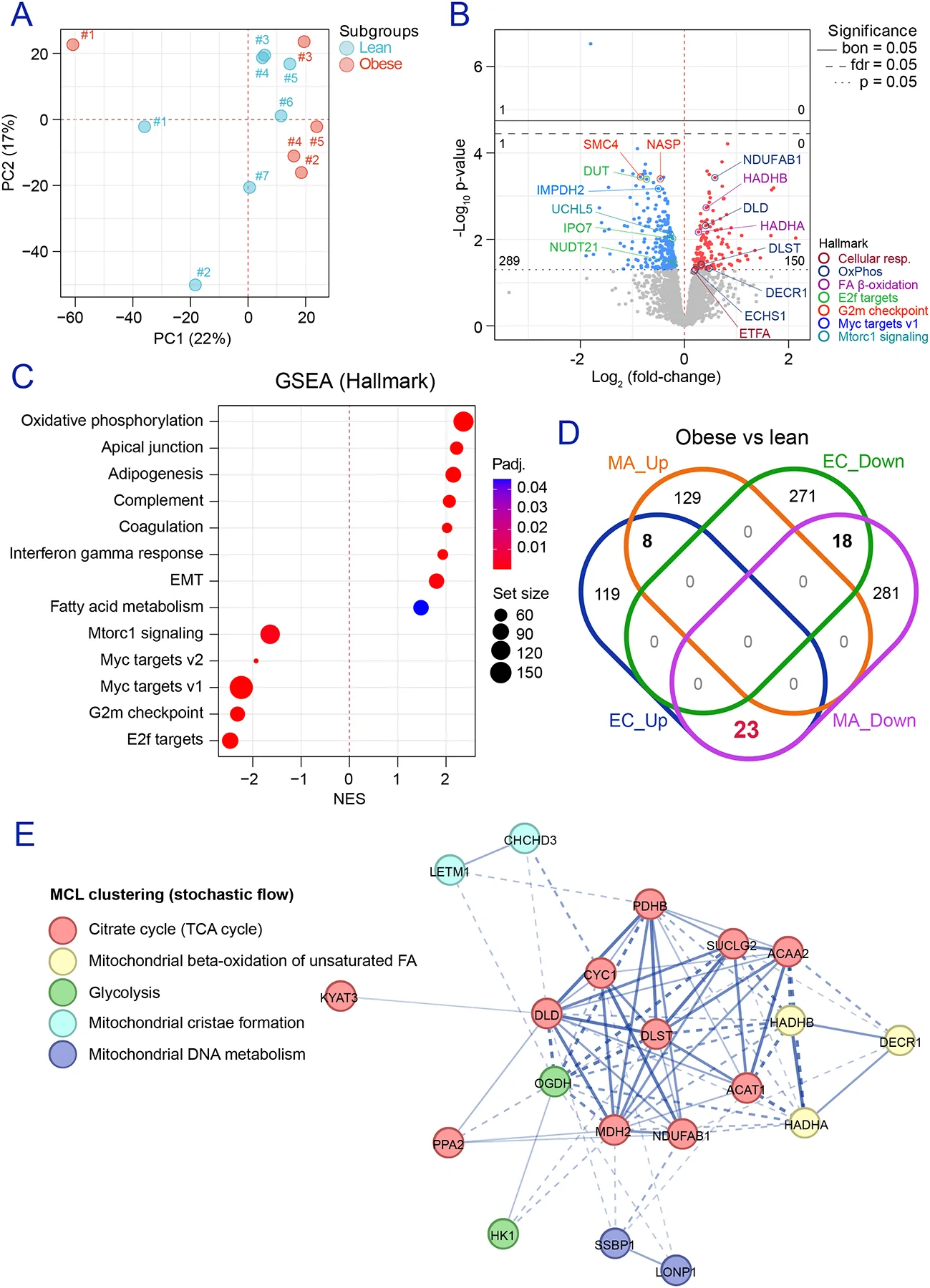
Mapping obesity-associated alterations in adipose-derived EC. **(a)** PCA shows the clustering of EC obtained in gallstone surgery patients with (BMI>30 kg/m^2^) and without (BMI<25 kg/m^2^) obesity. **(b)** The scatter dot plot shows DAPs up (red) and down-regulated (blue) in “obese” (n=5) vs “lean” (n=7) EC. Statistical significance was assessed by employing two-tailed Bayesian moderated t-tests and assuming equal variance in unpaired comparisons. Dots in grey show proteins meeting our exclusion criteria (nominal p-value≥0.05). Coloured labels point at protein landmarks of significance in relevant pathways. **(c)** GSEA (Hallmark) annotations revealed, among others, the downregulation of cell cycle-related pathways and the upregulation of OXPHOS, mitochondrial performance, and lipid handling in subjects with obesity. **(d)** Integration of protein datasets obtained in ex vivo isolated “obese” vs “lean” adipose-resident cells show an apparent number of proteins (23) with opposite sign in cultures of EC and isolated mature adipocytes (MA). **(e)** Gene concept network depicting the relationships between these 23 proteins. The Markov Cluster Algorithm (MCL) was used to find natural clusters based on the stochastic flow (inflation parameter: 5). Pathways with the same color correspond to the same cluster. Line thickness indicates the strength of data support. Dotted lines show edges between clusters. Source data is provided as a **Source data file**.

**Figure 3.**
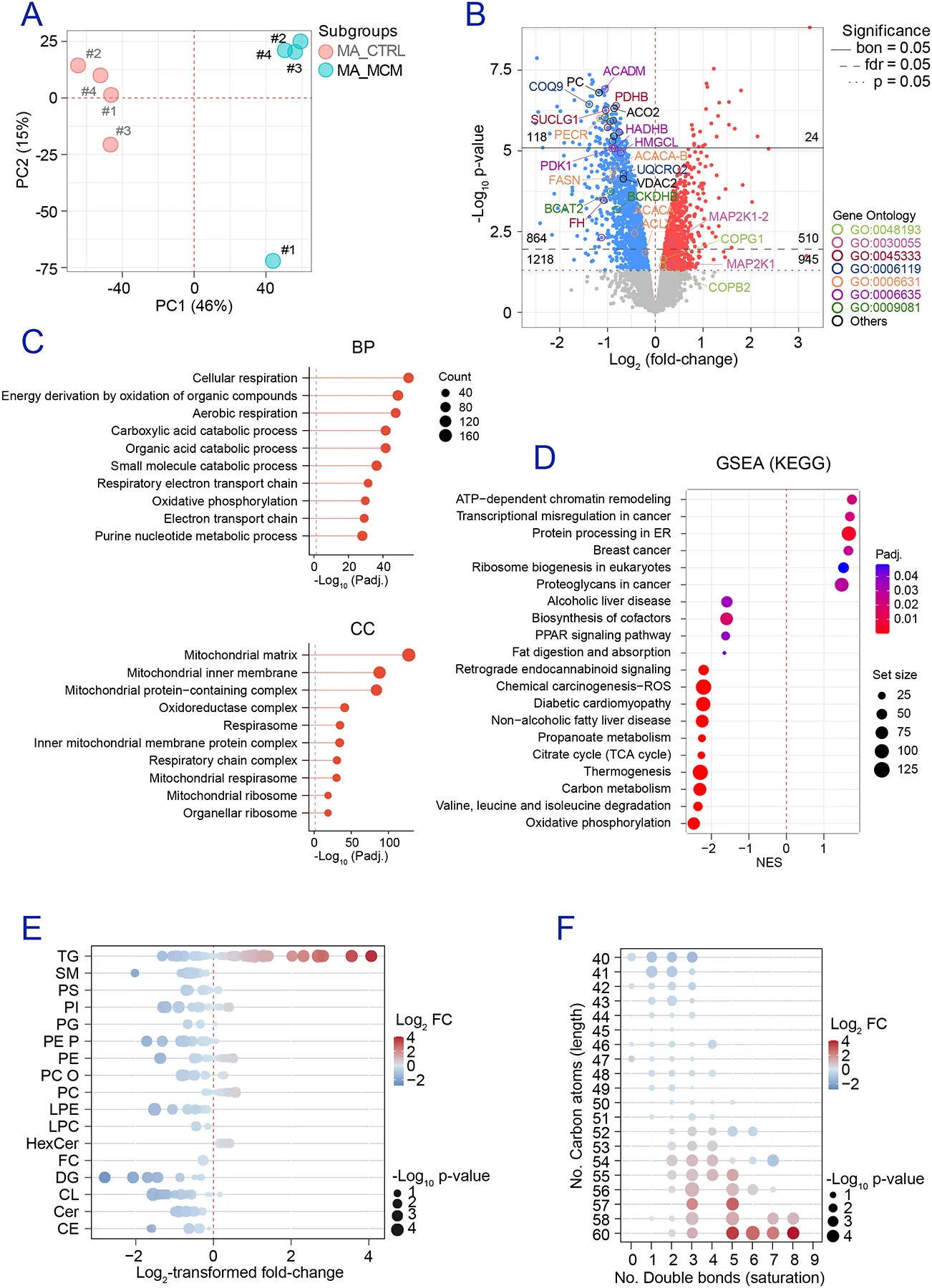
Macrophage-derived cytokines impact molecular patterns in adipocytes. **(a)** PCA shows the clustering of primary adipocyte cultures upon treatment with LPS-conditioned macrophage media (MCM) vs control (fresh macrophage media), n=4/group. **(b)** The volcano plot shows DAPs up (red) and down-regulated (blue) in MCM-activated adipocytes. Statistical significance was assessed by employing two-tailed Bayesian moderated t-tests and assuming equal variance in unpaired comparisons, and dots in grey show proteins meeting our exclusion criteria (nominal p-value≥0.05). Colored labels point at protein landmarks of GO pathways significantly regulated in activated cells, as detailed in Figure 1 legend. **(c)** ORA enrichment terms in a GO computational framework. Additional details are provided in **Table S9**. **(d)** GSEA (KEGG) annotations according to the main function of altered protein hubs. **(e)** Lipidomes conducted in control (n=3) and MCM-activated (n=3) adipose cells showed the modulation of many glycero- and sphingolipid species: TG, SM, phosphatidylserines (PS), phosphatidylinositols (PI), phosphatidylglycerols (PG), PE and PE based plasmalogens (PE P); PC and PC O; lysophosphatidylethanolamines (LPE), lysophosphatidylcholines (LPC), hexosylceramides (HexCer), FC, DG, cardiolipins (CL), ceramides (Cer), and cholesteryl esters (CE). The modulation of phosphatidic acids (PA) is not shown due to their very low amounts. Bubble plot in **(f)** shows TG species segregated according to the saturation (n° double bonds) and FA length (n° carbon atoms). Statistical significance was assessed by employing two-tailed Student’s t-test between study and control group. Differential abundance of lipids was identified based on an absolute log_2_ FC value≥0.58 and p-value<0.05. Source data for Figures 3c and 3d is provided as a **Source data file**.

**Figure 4.**
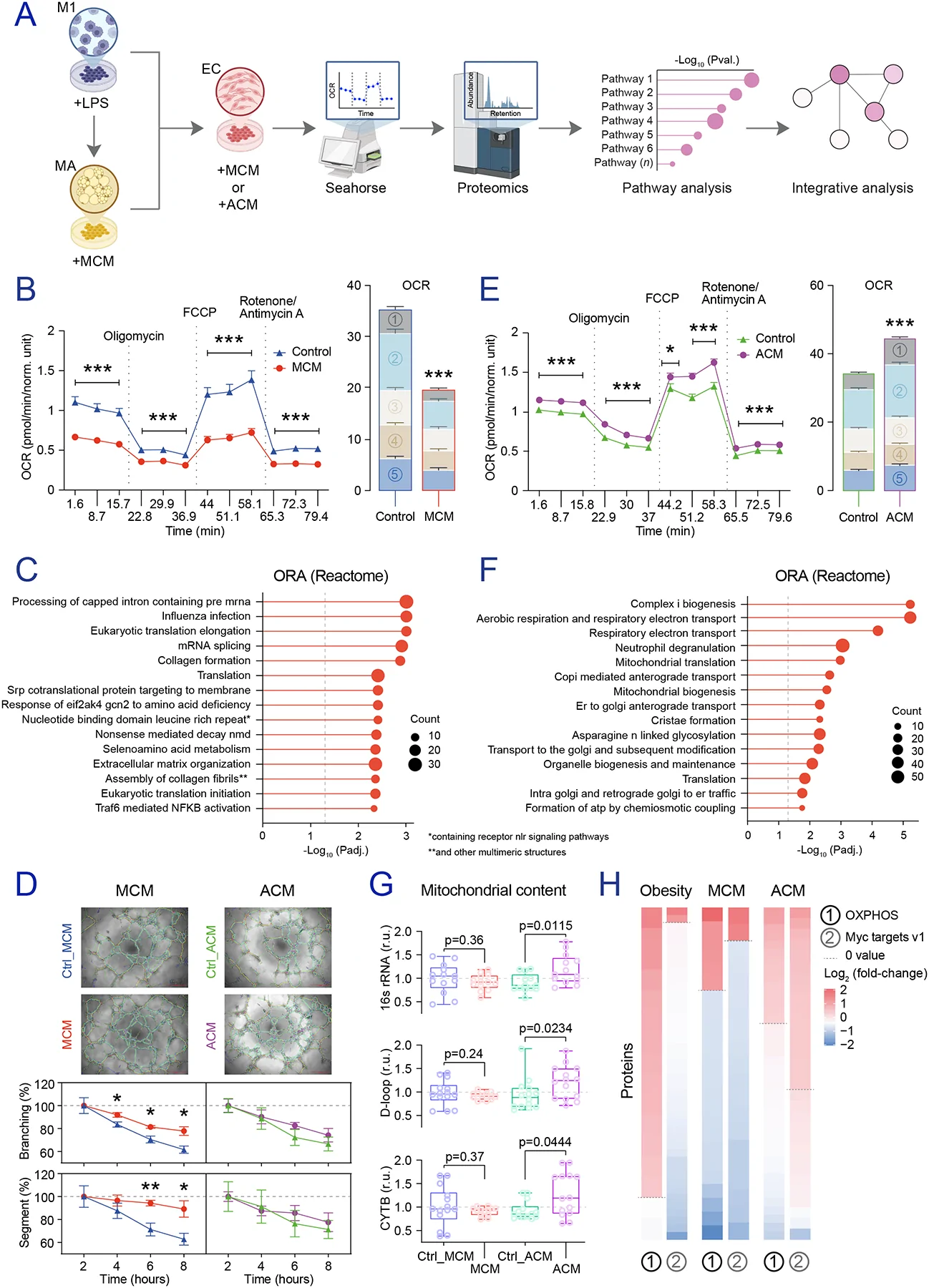
Macrophage and adipocyte-conditioned media trigger different EC responses. **(a)** Schematic representation of our workflow in HAMEC cultures. **(b)** Seahorse mitochondrial stress flux analysis and measures of oxygen consumption rate (OCR) in HAMEC cultures treated with 1% MCM for 72 hours (3 assays). **(c)** ORA of proteomic results (see in **Figure S6a-g**) and pathway interpretation according to the open-source, curated and peer-reviewed Reactome database. **(d)** Comparative network patterns of HAMEC and time-course network analysis according to the “Endothelial Tube Formation Assays” (ETFA). Phase contrast images with the superposition of vectorial objects were obtained from computer analysis using the customized “Angiogenesis Analyzer” for ImageJ. In the example provided (6 hours), in green, *Branches*; magenta, *Segments*; red surrounded by blue, *Junctions*; cyan, *Meshes*; violet, *Anchorage Junctions*; red circle, *Sphere*. ETFA scale bar, in red: 750 μm. **(e)** Seahorse mitochondrial stress flux analysis and OCR measures in HAMEC when treated with 10% ACM for 72 hours (3 assays). **(f)** ORA of proteomic results (see in **Figure S7a-g**), and pathway interpretation according to Reactome. **(g)** Analysis of mitochondrial components in MCM and ACM-activated HAMEC (3 assays). **(h)** The heatmap shows, in each dataset, the significant modulation of scoreboard proteins in OXPHOS and Myc targets-related biological processes (Hallmark). In Seahorse assays, (**1**) spare respiratory capacity, (**2**) maximal respiration, (**3**) basal respiration, (**4**) ATP-production coupled respiration, and (**5**) non-mitochondrial oxygen consumption. Mann-Whitney test (Holm-Šídák method for multiple comparisons). *p<0.05, **p<0.01, ***p<0.001. Source data is provided as a **Source data file**.

**Figure 5.**
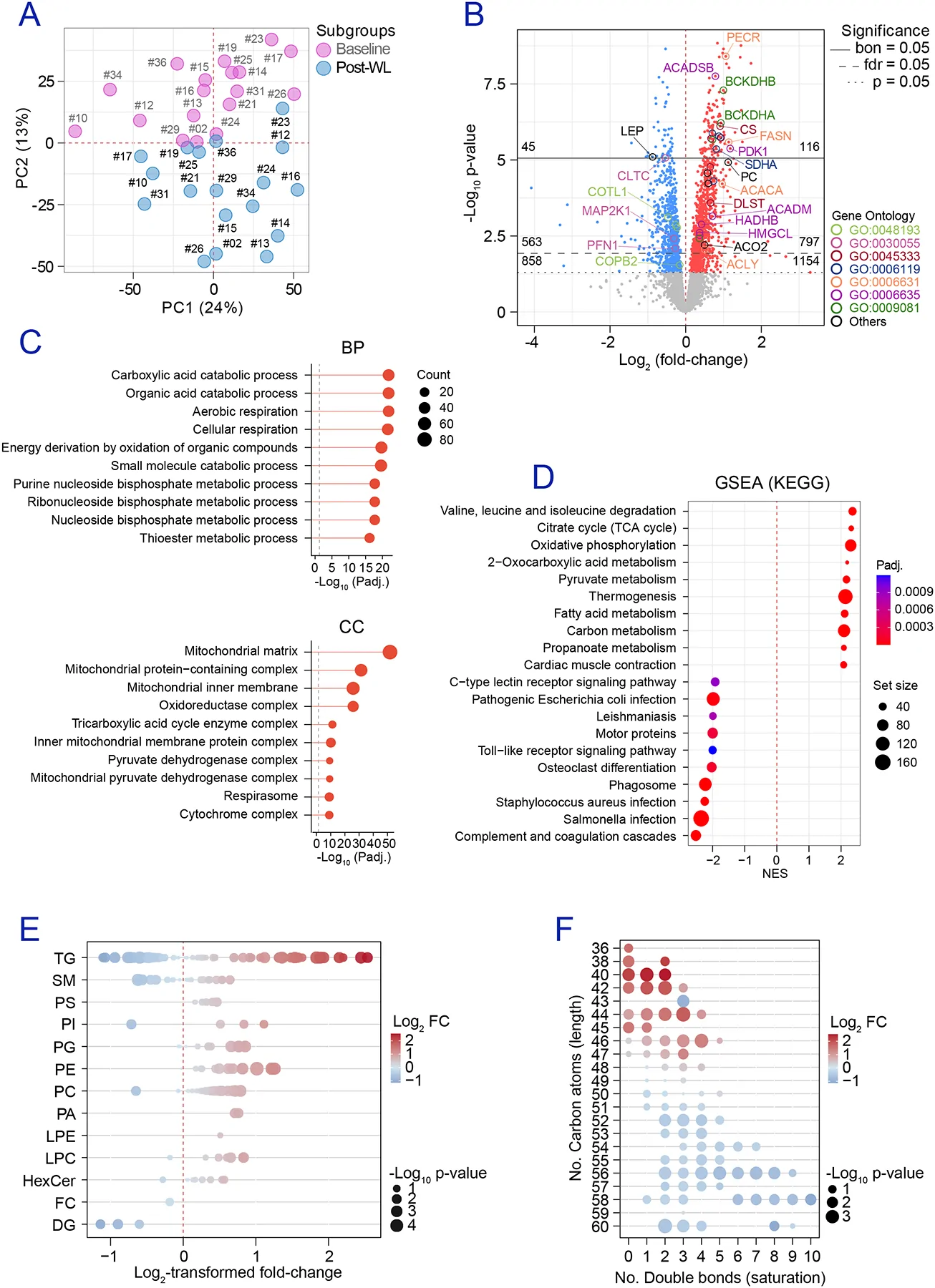
Weight loss reshapes adipose tissue protein and lipid landscapes. **(a)** PCA shows the clustering of fat samples obtained at baseline and following weight loss (WL) (n=18). **(b)** The volcano plot shows DAPs up (red) and down-regulated (blue) in subcutaneous adipose tissue after WL. Statistical significance was assessed by employing two-tailed Bayesian moderated t-tests, assuming equal variance in paired comparisons. Dots in grey show proteins meeting our exclusion criteria (nominal p-value≥0.05). Coloured labels point at protein landmarks of GO pathways, as detailed in Figure 1. **(c)** Bubble plots created by applying ORA to enrichment terms in a GO computational framework. ORA pathways were segregated in Biological processes (BP) and Cellular components (CC). Additional details are provided in **Table S12**. **(d)** Gene set enrichment analysis (GSEA) and Kyoto encyclopaedia of genes and genomes (KEGG) annotations confirmed the upregulation of mitochondrial processes mostly related to fatty acid metabolism, running together with the downregulation of immune response determinants after WL. **(e)** The dynamic nature of lipids in bulk adipose tissue before-after WL (n=20) pointed at the modulation of a number of glycerophospholipid and sphingolipid species, including TG, SM, PS, PI, PG, PE, PC, PA, LPE and LPC; HexCer, FC, and DG. Bubble plot in **(f)** shows modulation of TG species, segregated according to the degree of saturation (n° double bonds) and length (n° carbon atoms) of fatty acids (FA). Lipid concentrations were normalized based on the total amount within each lipid class, followed by log_2_ transformation for conducting two-tailed Student’s t-test between study and control groups. Differential abundance of lipids was identified based on an absolute log_2_ fold-change (FC) value≥0.58 and nominal p-value<0.05. Source data for Figures 5c and 5d are provided as a **Source data file**.

**Figure 6.**
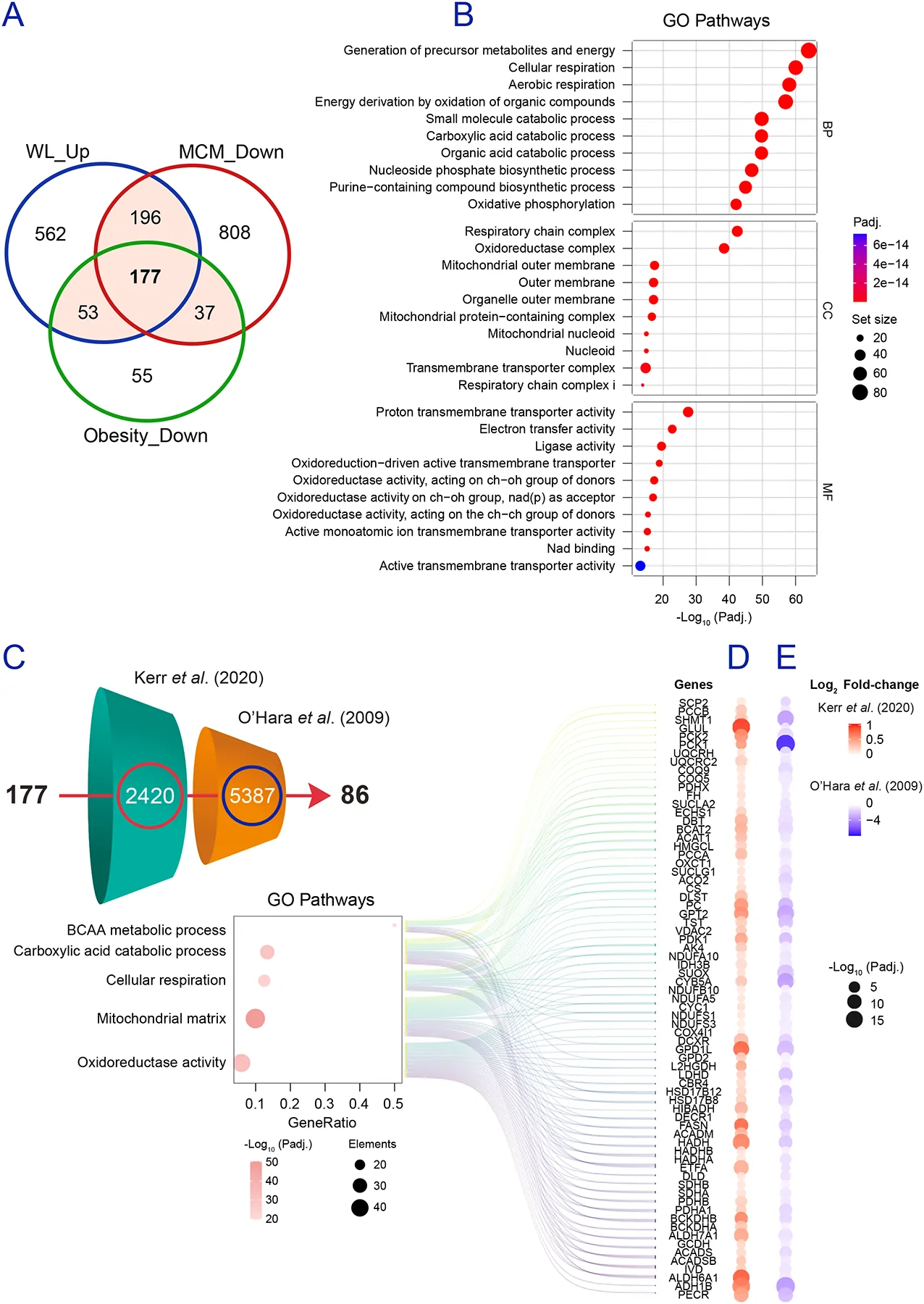
Restoration of fat metabolism after weight loss mostly lies in adipocytes. **(a)** The Venn diagram shows the number of proteins that are upregulated in bulk adipose tissue after WL (blue circle), but decreased in adipocyte cultures when challenged with macrophage-derived cytokines (red circle), and in “obese” when compared to “lean” isolated adipocytes (green circle). For the selection we considered proteins showing unadjusted two-tailed p-values<0.05 in Bayesian moderated t-tests. Common elements are represented by the intersections of circles. The intersection faintly inked shows 177 conjointly regulated proteins. The pathways underlined according to the ORA (GO) are provided in **(b)**. The funnel diagram in **(c)** depicts the comprehensive pipeline used to shortlist potential target proteins (genes) across independent transcriptional studies in bulk fat after surgery-induced WL [55] and experiments in MCM-inflamed SGBS-derived adipocytes [56] (see in **Figure S10a** for further detail on our experimental framework). Bubble plots in **(d)** and **(e)** shows how these gene candidates behave in those alternative datasets, and the pathways they belong to. Source data are provided as a **Source data file**.

**Figure 7.**
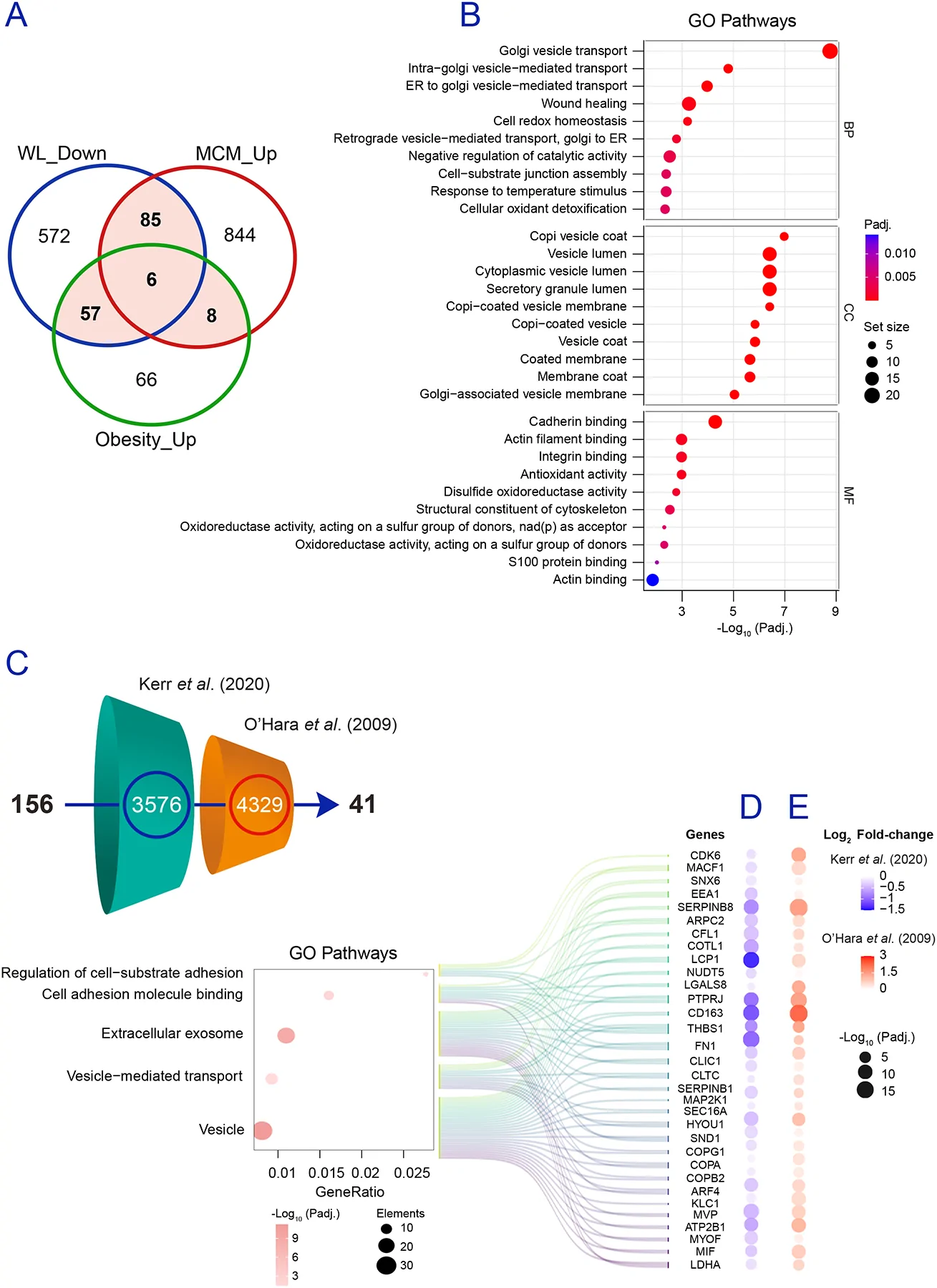
Increased adipocyte vesicle transport and ECM adherence in obesity. **(a)** The Venn diagram shows the number of proteins that are coincidentally downregulated in adipose tissue after WL (blue circle), but increased in inflamed adipocyte cultures (red circle), and “obese” adipocytes (green circle). For the selection we considered proteins with unadjusted two-tailed p-values<0.05 in Bayesian moderated t-tests. The colored intersections include a total of 156 proteins showing at least 2 over 3 matches, when considering together our results in vivo, in vitro, and ex vivo. The pathways represented in this list of proteins are listed in **(b)**. The pipeline in **(c)** led us to cut down to 41 the gene (protein) candidates significantly associated, also at the transcriptional level, with **(d)** WL and **(e)** adipocyte inflammation. See in **Figure S10a** for further detail on our experimental workflow. Source data are provided as a **Source data file**.

**Figure 8.**
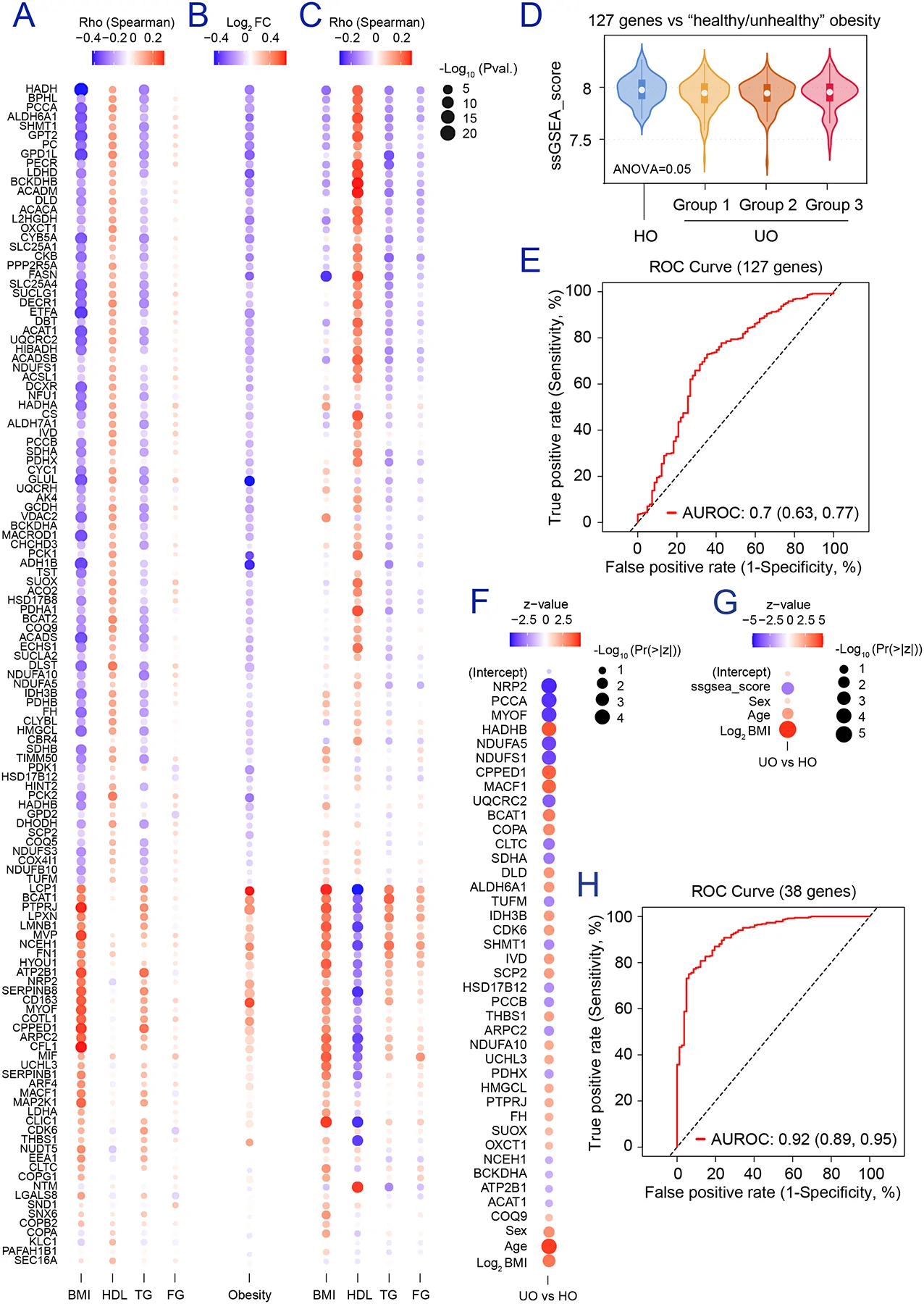
Gene association with obesity-related metabolic outcomes. Bubble plots in **(a)** (METSIM [22]) and **(c)** (ADIPOMIT [24]) show in cross-sectional studies Spearman’s rank correlation coefficients (Rho) and two-tailed p-values for correlations between metabolic traits (i.e., BMI, HDL, TG, and fasting glucose) and the expression of our 127 gene candidates in adipose tissue. Bubble plot in **(b)** (Finnish Twin Cohort [23]) show the Log_2_ fold-change (FC) and false discovery rate (FDR) p-values, when comparing the expression of these genes in the adipose tissue of monozygotic twin pairs with vs without obesity. **(d)** The violin plot and **(e)** ROC curve show the ability of this 127 gene cluster to segregate “healthy” (HO) and “unhealthy” (UO) obesity. Groups 1 to 3 indicate the number of metabolic comorbidities (i.e., dyslipidaemia, hypertriglyceridemia, and/or hyperglycaemia) observed, together with obesity, in ADIPOMIT participants. Bubble plots **(f)** and **(g)** show the stepwise regression model built upon our 127 gene candidates, along with sex, age and BMI as covariates, to stablish the final model of 38 genes, **(h)** able to better pick up the appearance of metabolic disturbances defining UO. See in **Figure S10b** for further details on our experimental workflow. Source data are provided as a **Source data file**.

**Figure 9.**
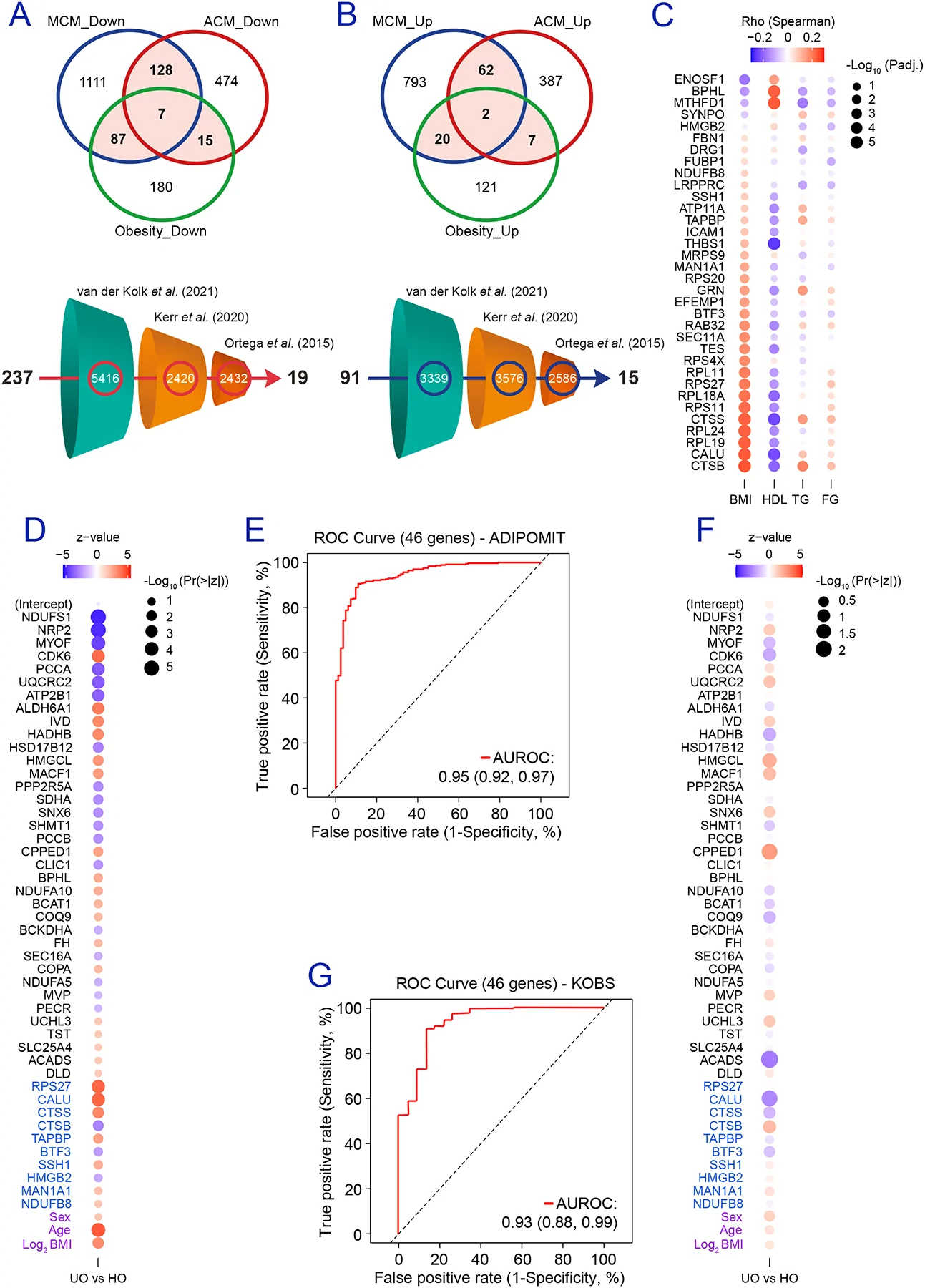
Adipocyte and EC proteins defining obesity-related meta-inflammation. Venn diagrams show the number of proteins that are coincidentally **(a)** down or **(b)** upregulated in MCM (blue circle) and ACM (red circle)-activated HAMEC, and in “obese” vs “lean” EC (green circle). For the selection we considered proteins with unadjusted p-values<0.05 in two-tailed Bayesian moderated t-tests. The coloured intersections highlight the number of proteins showing ≥2 over 3 potential matches, when considering together our results in vitro, in cells derived from fat biopsies, and in vivo. Funnel diagrams show the pipeline we followed to cut down the gene (protein) candidates associated, also at the transcriptional level, with WL in three independent bulk adipose tissue transcriptomes. Bubble plot in **(c)** shows Spearman’s rank correlation coefficients (Rho) and two-tailed p-values for correlations between BMI, HDL, fasting TG, and blood glucose and the expression of our 34 gene candidates in the obese population of the ADIPOMIT study. See in **Figure S11a** for further detail on our experimental framework. Bubble plot in **(d)** shows the stepwise regression model built upon our proteomic-transcriptomic results, along with sex, age, and BMI as covariates, to stablish the final model of 46 genes, able to better pick up the appearance of metabolic disturbances defining UO, as shown in **(e)**. Next, bubble plot in **(f)** and ROC curve in **(g)** show the performance of our curated, final 46-gene signature in dissociating “healthy” (HO) from “unhealthy” (UO) obesity also in the KOBS cohort. Source data are provided as a **Source data file**.

**Figure 10.**
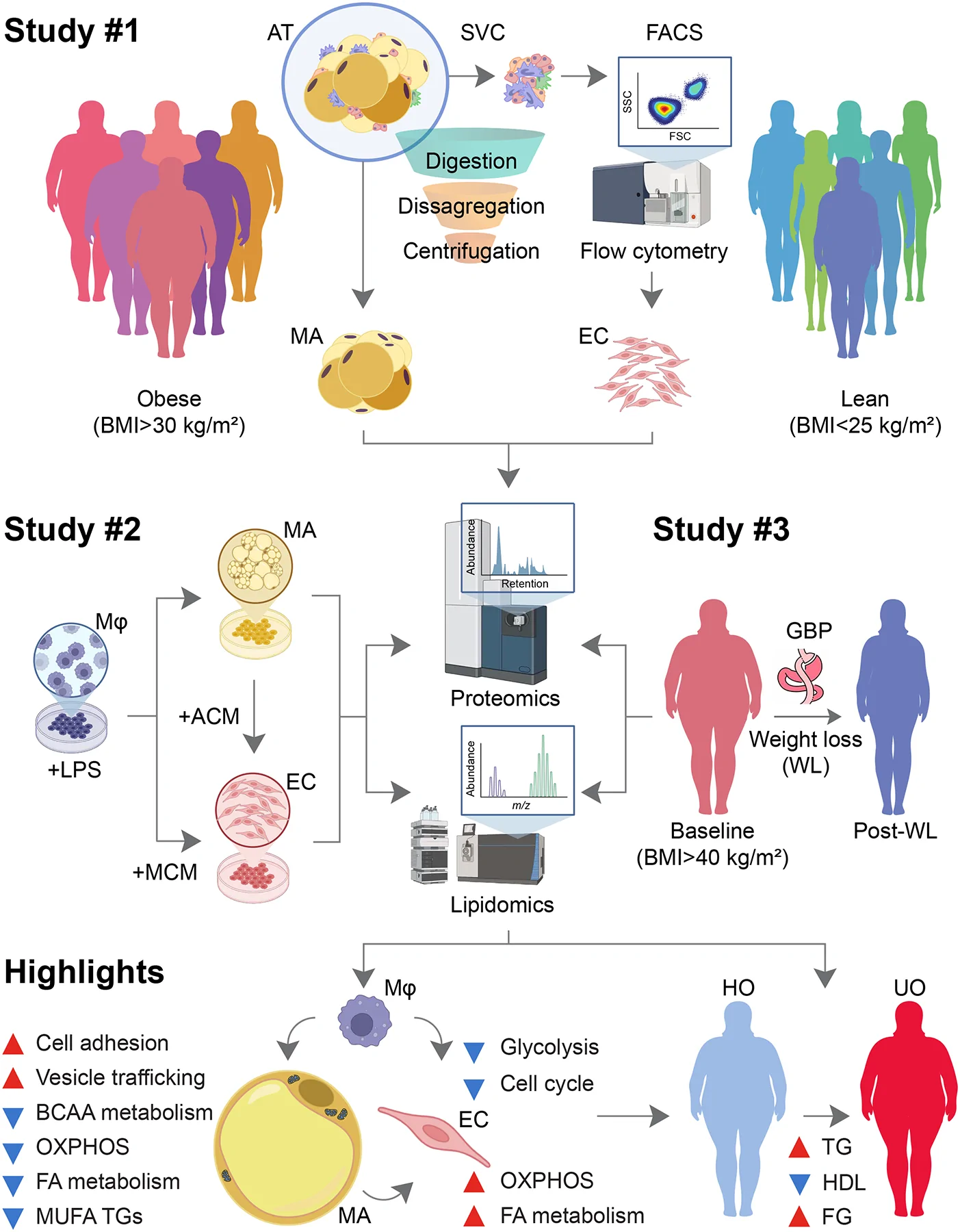
An adipose non-immune cell-type-informed, cross-context framework to identify molecular signatures of metabolic vulnerability in obesity. Here, we used a layered design to **(Study #1)** define, in human adipose tissue (AT), obesity-associated, cell-type-resolved molecular phenotypes in both mature adipocytes (MA) and endothelial cells (EC) within stromal vascular cells (SVC), **(Study #2)** mimic an obesity-like inflammatory milieu and probe adipocyte-to-endothelial signaling using macrophage (MCM) and adipocyte-conditioned media (ACM), and **(Study #3)** determine which obesity-associated features show evidence of recovery after surgery-induced weight loss (GBP) in bulk fat. We then curated a robust gene signature by intersecting obesity and inflammation-associated candidates with multiple transcriptomic datasets, and evaluated physiological relevance across human cohorts, including derivation of a refined gene signature capable of dissociating “healthy” (HO) from “unhealthy” (UO) obesity. Thereby, our study provides a comprehensive resource model for adipose cell-specific determinants acting as predictors of metabolic vulnerability in people living with obesity.

## Data Availability

The source data underlying the findings of this study has been deposited in public data repositories, and/or is presented as a Source Data file with the manuscript. Protein raw data, processing and statistical analysis can be accessed at the ProteomeXchange Consortium via the MassIVE partner repositories (http://proteomecentral.proteomexchange.org) with the following identifier: PXD070733 (MassIVE ID: MSV000099905), under the accession DOI number 10.25345/C54Q7R37G. The lipidomic data of human adipose tissue and ex vivo isolated adipocytes has been made publicly available in the Figshare repository, under accession DOI number 10.6084/m9.figshare.28638761. Data on human preadipocyte cultures differentiated into adipocytes and treated with MCM-induced inflammatory conditions is available under accession DOI number 10.6084/m9.figshare.25541005. Additional datasets used during this research are publicly available in the GEO repository under the following accession codes: GSE199063 [55], GSE14312 [56], and GSE53378 [60].
